# Transcriptional and Epigenetic Consequences of DMSO Treatment on HepaRG Cells

**DOI:** 10.3390/cells11152298

**Published:** 2022-07-26

**Authors:** Hélène Dubois-Pot-Schneider, Caroline Aninat, Kathrin Kattler, Karim Fekir, Kathleen Jarnouen, Virginie Cerec, Denise Glaise, Abdulrahman Salhab, Gilles Gasparoni, Kubo Takashi, Seiichi Ishida, Jörn Walter, Anne Corlu

**Affiliations:** 1 INSERM, Université de Rennes, INRAE, Institut NuMeCan (Nutrition, Metabolisms and Cancer), F-35000 Rennes, France; caroline.aninat@univ-rennes1.fr (C.A.); karim.fekir@gmail.com (K.F.); kathleen.jarnouen@live.fr (K.J.); v.cerec@gmail.com (V.C.); denise.glaise@free.fr (D.G.); anne.corlu@inserm.fr (A.C.); 2 Department of Genetics, University of Saarland (UdS), 66123 Saarbrücken, Germany; kathrin.kattler@uni-saarland.de (K.K.); abdulrahman.salhab@uni-saarland.de (A.S.); gillesgasparoni@googlemail.com (G.G.); j.walter@mx.uni-saarland.de (J.W.); 3 Division of Pharmacology, National Institute of Health Sciences, Kawasaki-ku, Kawasaki 2109501, Japan; tkubo@ncc.go.jp (K.T.); ishida-s@bio.sojo-u.ac.jp (S.I.)

**Keywords:** DMSO, HepaRG cells, hepatic differentiation, gene expression, histone modification

## Abstract

Dimethyl sulfoxide (DMSO) is used to sustain or favor hepatocyte differentiation in vitro. Thus, DMSO is used in the differentiation protocol of the HepaRG cells that present the closest drug-metabolizing enzyme activities to primary human hepatocytes in culture. The aim of our study is to clarify its influence on liver-specific gene expression. For that purpose, we performed a large-scale analysis (gene expression and histone modification) to determine the global role of DMSO exposure during the differentiation process of the HepaRG cells. The addition of DMSO drives the upregulation of genes mainly regulated by PXR and PPARα whereas genes not affected by this addition are regulated by HNF1α, HNF4α, and PPARα. DMSO-differentiated-HepaRG cells show a differential expression for genes regulated by histone acetylation, while differentiated-HepaRG cells without DMSO show gene signatures associated with histone deacetylases. In addition, we observed an interplay between cytoskeleton organization and EMC remodeling with hepatocyte maturation.

## 1. Introduction

The liver plays a major role in xenobiotic and energetic metabolisms and is involved in a great number of functions including plasma protein synthesis, bile production, iron metabolism, and immune surveillance. Thus, as an organ with pleiotropic functions, the liver is associated with many types of diseases including hepatitis, cholestatic diseases, metabolic disorders, acute and chronic failure, cirrhosis, and carcinomas. With its drug-metabolizing function, it is also the major site of drug-induced injury. To study the metabolism and potential hepatotoxicity of chemicals, pharmaceutical companies and biotech industries are currently interested in relevant in vitro screening or testing models that evaluate the safety of candidate compounds accurately and effectively, that allow reducing animal drug testing and costly late-stage failures during pharmaceutical clinical trials. As hepatocytes are the major cell constituent of the liver and play an important role in the detoxification of xenobiotics, many culture models have been developed in an effort to relate in vitro findings to in vivo ones in order to study xenobiotic metabolism [1,2,3]. Although expression levels and induction activities of drug-metabolizing enzymes are far from those found in hepatocytes, established cell lines mainly originated from tumors, such as HepG2, are extensively used. These cell lines lack a variable and substantial set of liver-specific functions, making them unsuitable as representative of in vivo liver parenchymal cells [4,5]. Therefore, until now, primary human hepatocytes (PHHs) remain the gold standard system for xenobiotic metabolism and toxicity studies. They are available and widely used in the field of hepatotoxicity testing. However, their use presents some defects. Indeed, differences in cell viability between batches of hepatocytes, rapid decrease in functionality after their isolation, and variation due to the inter-individual differences of donors have been described [6,7]. New in vitro models are still under development or characterization. Embryonic stem cells (ES) induced pluripotent stem cells (iPS), liver stem cells, or mesenchymal stem cells (MSC)-derived hepatocytes could be promising tools to propose further a suitable alternative to PHHs. However, although these stem cells have been shown to be able to differentiate toward hepatocytic lineage under defined culture conditions, to repopulate the liver and complete their maturation in vivo, they are not enough functional to allow drug metabolism studies and disease modeling in vitro [8,9,10,11,12]. Recently, three-dimensional culture protocols have improved the differentiation of stem cells in hepatocyte-like cells. However, the cells still co-express progenitor and hepatocyte markers showing that their phenotype remains comparable to human neonate hepatocytes [13,14]. Among all in vitro models, differentiated HepaRG cells are currently the closest to PHHs [5,7,15,16,17]. HepaRG cells derived from a human hepatocellular carcinoma maintain activities of many metabolizing enzymes [4,18,19] and therefore provide a valuable in vitro model for human drug metabolism studies in normal or steatotic conditions [20,21,22,23].

Methods to stabilize the liver-specific functions of hepatocytes in vitro as well as differentiation of ES cells, iPS and mesenchymal cells involve growth factors, hormones, such as insulin and glucocorticoids, extracellular matrixes, or co-culture with non-parenchymal cells [24,25,26]. The dipolar aprotic solvent dimethyl sulfoxide (DMSO) is also wildly used to sustain liver-specific gene expression [27]. DMSO was first used by Isom et al. in combination with a collagen-coated surface in an attempt to extend the time that rat hepatocytes remain differentiated in vitro [28,29]. In these conditions, DMSO increases gap junction-mediated intercellular communication between mouse or rat hepatocytes [30,31]. It favors the maintenance of normal hepatic transcription factor patterns, basal cytochrome P450, and nuclear receptor profiles as well as lipid synthesis and secretion [32,33]. Finally, it mediates apoptosis inhibition through an efficient inactivation of cleaved initiator caspases and prevention of ASK1-JNK activities via GST regulation [34]. In the hepatoma cell line Huh-7, DMSO exposure arrests the cell cycle in G0/G1 state and increases the cell differentiation status including drug metabolism [35,36]. In HepG2 cells, DMSO incubation changes lipid and glucose metabolisms, which become closer to the PHH ones [37]. More recently, DMSO was included in protocols of hepatocyte differentiation from human ES, mouse iPS, and MSC [8,38,39,40,41]. It has been first shown that priming hESC differentiation to definitive endoderm prior to DMSO treatment would be crucial in leading to more efficient hepatocyte differentiation [40]. Then, it has been observed that the addition of DMSO in the initial stage of definitive endoderm specification induces rapid downregulation of pluripotent genes, restored checkpoint control of the cell cycle preparing proliferation and transition to differentiated phenotypes [42,43].

The underlying mechanisms by which DMSO exerts its effect on cell survival and differentiation are still in need of further investigation. Several studies reveal DMSO-associated alterations on the epigenetic level, in particular histone modifications [44,45,46]. For instance, while DMSO treatment of mouse 2-cell embryos resulted in an overall reduction in protein acetylation, increased histone H3 and H4 acetylation were observed along with impaired transcriptional programs [44]. On the other hand, a recent study in HepG2 showed that the DMSO-induced increase in CYP3A4 expression is mediated by a cascade of histone modification changes [46]. While these studies raise intriguing possibilities of a histone modification mediated DMSO effect on transcriptional programs, to date systematic genome-wide analyses are missing.

Thus, we used the HepaRG human hepatoma cell line [47] to study the effect of DMSO on transcriptional and epigenetic programs during hepatic differentiation. HepaRG cells exhibit a pseudodiploid karyotype and share with the human fetal liver multipotent progenitor cells isolated and characterized by Dan et al., a high proliferative ability, a differentiation potential toward biliary and hepatocytic lineage, and the capacity to differentiate into functional hepatocytes [18,19,48,49]. Although hepatic differentiation occurs spontaneously in this cell line, the addition of DMSO during the differentiation process improves the infectability of HepaRG cells by Hepatitis B virus and the expressions of liver-specific genes, including drug-metabolizing enzymes, which reach levels close to those found in PHHs [7,18,22,47,50]. Thus, compared to other hepatoma cell lines, HepaRG cells provide a unique tool for identifying DMSO mechanisms favoring hepatic gene expression. Few studies have already been performed to analyze the impact of DMSO addition on HepaRG cells [51]. Only a small set of genes (<50) related to metabolism and detoxification function has been identified in link with the DMSO although large-scale analyses focused on HepaRG differentiation are available [5,52].

In this context, we performed a large-scale analysis to determine the global role of DMSO exposure during the differentiation process. As evaluations of drug toxicity are usually performed with a lower level of DMSO or after DMSO removal [53,54,55] we also assessed the effect of DMSO removal after differentiation.

## 2. Materials and Methods

### 2.1. Chemicals

LY294002, an inhibitor of PI3K (Tebu-bio, Le Perrayen Yvelines, France) was used at 10 µM. Colchicine (Merck, C3915) was used at 10 µM. DMSO was purchased from Sigma (St. Quentin Fallavier, France, D4540).

### 2.2. Cell Culture

HepaRG cell line was maintained (passage number 8–12) as previously described [47,48]. Cells were seeded at a density of 2.7 × 104/cm^2^ and maintained for two weeks in a William’s E medium supplemented with 10% fetal bovine serum, 100 U/mL penicillin, 100 μg/mL streptomycin, 5 μg/mL insulin, and 50 μM hydrocortisone. Then, the culture medium was or was not supplemented with 2% DMSO for two additional weeks. Cells were usually collected at this stage (D30 − DMSO, D30 + DMSO). To evaluate the impact of DMSO removal, it was eliminated from differentiated HepaRG culture at day 30 (D30 + DMSO) and cells were collected 4 days later (D34 ± DMSO) (Figure S1). For LY294002 and colchicine treatments, HepaRG cells were exposed at day 15 with 10 µM LY294002 or 2% DMSO + 10 µM colchicine for 24 h.

### 2.3. Real-Time RT-PCR

Total RNA was purified with an RNAeasy kit (Qiagen, Valencia, CA, USA). cDNA was obtained by using the High-Capacity cDNA Reverse Transcription Kit (Applied Biosystems Waltham, MA, USA) according to manufacturer’s instructions. The Real-time PCR was performed with The StepOnePlus™ System and the fluorescent dye SYBR Green methodology using the SYBR Green PCR Master Mix (Applied Biosystems). The expression was normalized with the housekeeping gene TBP (TATA-binding protein). Primer sequences are listed in Table S1. For TDO2 the real-time PCR was performed with the Taqman probe-based assays (Applied Biosystems). Human Taqman primers for PCK2 (Hs00388934_m1), TDO2 (Hs00194611_m1), and the housekeeping gene TBP (Hs00427620_m1) were provided by Applied Biosystems. Data were quantified with the StepOne Plus software v2.2.1.

### 2.4. Microarray Analysis

mRNAs from four independent biological replicates of each time point were used to perform the microarray experiment. Genome-wide expression profiling was performed using the low-input QuickAmp labeling kit and human SurePrint G3 8x60K pangenomic microarrays (Agilent Technologies, Santa Clara, CA, USA). Gene expression data were processed using Feature Extraction (Agilent Technologies). The data discussed in this publication have been deposited in NCBI’s Gene Expression Omnibus (GEO, RRID:SCR_005012) and are accessible through GEO Series accession number GSE112123 since 25 July 2022 (https://www.ncbi.nlm.nih.gov/geo/query/acc.cgi?acc=GSE112123). Statistical analyses were performed with GeneSpring GX software v11.5 (Agilent Technology Santa Clara CA USA). Differentially expressed genes along the differentiation were identified by a one-way ANOVA test (with a Bonferroni FWER correction) with a *p*-value < 0.001 and an absolute fold change (|FC|) > 3. Clustering analysis was performed using Cluster 3.0 and TreeView 1.6 (Java Treeview, RRID:SCR_016916) using uncentered correlation and complete linkage. Gene annotation was based on gene ontology and enrichment for specific biological functions was determined using FuncAssociate 2.0 program (FuncAssociate: The Gene Set Functionator, RRID:SCR_005768) [56]. To determine genes significantly deregulated between two conditions (D30 + DMSO vs D30 − DMSO or D34 ± DMSO vs D30 + DMSO), a *t*-test (with Benjamini-Hochberg correction) with a *p*-value < 0.005 and a |FC| > 2 was used. Genes were considered as stable between two conditions (D30 + DMSO vs D30 − DMSO or D34 ± DMSO vs D30 + DMSO) when they were deregulated during HepaRG differentiation (ANOVA of all conditions, *p* < 0.01; |FC| > 2) but had a coefficient of variation less than 30% (%CV < 30%) and |FC| < 1.3 in both compared conditions. 

### 2.5. Ingenuity Pathways Analysis

Upstream regulators were identified using Ingenuity Pathway Analysis Tool (IPA, Ingenuity Pathway Analysis, RRID:SCR_008653) based on the downregulated, upregulated or stable gene lists.

### 2.6. Gene Set Enrichment Analysis

Gene set enrichment analysis (GSEA, Gene Set Enrichment Analysis, RRID:SCR_003199) was used to check whether an a priori defined set of genes shows statistically significant, concordant differences between two biological states. GSEA was performed by using the Java tool developed at the Broad Institute (Cambridge, MA, USA). Unsupervised GSEA was performed with the whole C2 collection of curated gene sets from the molecular signatures database (MSigDB). Enrichment score (ES) was determined after 1,000 permutations, as previously described [57,58].

### 2.7. Connectivity Map

Connectivity map (Connectivity Map 02, RRID:SCR_015674) algorithm was used to link gene expression signatures with putative therapeutic molecules [59].

### 2.8. Chromatin Immunoprecipitation Followed by Next Generation Sequencing (ChIP-seq)

Detached HepaRG cells were fixed with 1% formaldehyde in William’s E medium for 10 min. Crosslinking was stopped by adding glycine to a final concentration of 125 mM for 5 min at room temperature. If not mentioned otherwise samples were thereafter kept on ice or at 4 °C and centrifugations were performed at 800 g for 5 min. After washing twice with 125 mM glycine in PBS and once in PBS, cells were resuspended consecutively in 5 mL buffer A (10 mM HEPES-NaOH, 10 mM EDTA, 0.5 mM EGTA, 0.25% Triton X-100, 1 X PIC (Complete Protease Inhibitor Cocktail Tablets)), respectively, buffer B (10 mM HEPES-NaOH, 200 mM NaCl, 1 mM EDTA, 0.5 mM EGTA, 0.01% Triton X-100, 1 X PIC) and rotated for 10 min followed by centrifugation each. Cells were resuspended in 600 μL buffer C (25 mM Tris-HCl, 150 mM NaCl, 2 mM EDTA, 1% Triton X-100, 0.25% SDS, 1 X PIC). Sonication of chromatin into fragments between 100 and 800 bp was performed using the Bioruptor NGS (Diagenode, Liège, Belgium) on high power setting in 200 μL volumes, either in 0.5 mL shearing tubes with 30 cycles or in 1.5 mL microtubes (Diagenode, Liège, Belgium) with 10 cycles and each cycle consisting of 30 s sonication and 30 s recovery time. Samples were diluted with double volume dilution buffer (25 mM Tris-HCl (pH 8.0), 150 mM NaCl, 1 mM EDTA, 1% Triton X-100, 0.75% Glycerol).

For each immunoprecipitation 10 µL protein G beads (Invitrogen, Carlsbad, USA) were combined either with 2 μg H3K4me3 (Diagenode Cat# pAb-003-050, RRID:AB_2616052), H3K27ac (Diagenode Cat# C15410196, RRID:AB_2637079) or H3K4me1 (Diagenode Cat# C15410194, RRID:AB_2637078) antibody, 5 μL BSA (NEB, Frankfurt am Main, Germany, 10 mg/mL) and rotated for 2 h. Then, 300 μL sonicated chromatin (equivalent to 1 million cells) was added. Immunoprecipitations of H3K27ac were supplemented with Na-Butyrate to a final concentration of 10 mM. After rotation overnight beads were consecutively washed with 1 mL of WB1 (20 mM Tris-HCl, (pH 8.0), 150 mM NaCl, 2 mM EDTA, 1% Triton X-100, 0.25% SDS), twice WB2 (20 mM Tris-HCl (pH 8.0), 500 mM NaCl, 2 mM EDTA, 1% Triton X-100, 0.25% SDS), WB3 (10 mM Tris-HCl, (pH 8.0), 250 mM LiCl, 1 mM EDTA, 0.5% Nonident P-40, 0.5% sodium deoxycholate) and twice TE/NaCl (10 mM Tris-HCl (pH 8.0), 50 mM NaCl, 1 mM EDTA) under rotation for 10 min. Elution was performed using 200 µL EB (100 mM NaHCO_3_, 1% SDS) for 30 min in a shaking device at room temperature. Eluates were supplemented with crosslink reversal solution (4 μL 5 M NaCl, 2 μL 0.5 M EDTA and 2.5 μL Proteinase K (20 mg/mL)) and incubated overnight at 55 °C. ChIP DNA was purified by phenol chloroform extraction followed by ethanol precipitation. Library preparation was performed using the Truseq ChIP Sample Preparation Kit (Illumina, San Diego, CA, USA) following the manual except for omitting size selection. All ChIP-seq libraries were sequenced 2 × 100 bp paired-end on a HiSeq2500 (Illumina, San Diego, CA, USA). The raw data discussed in this publication have been deposited in NCBI’s Gene Expression Omnibus (GEO, RRID:SCR_005012) and are accessible through GEO Series accession number GSE179988 since 25 July 2022 (https://www.ncbi.nlm.nih.gov/geo/query/acc.cgi?acc=GSE179988). 

### 2.9. ChIP-seq Data Analysis

Low quality ends (phred score = 20) of FastQ format reads were trimmed in addition to adapter removal using Trim Galore (version 0.3.3) [http://www.bioinformatics.babraham.ac.uk/projects/trim_galore/ accessed on 13 May 2022]. Trimmed reads were aligned to the human reference genome (hs37d5) using GEM mapper (version 1.376 beta) [60]. Samtools (version 1.3) was used to convert SAM to BAM format [61]. MarkDuplicate (version 1.115) from Picard tools [http://broadinstitute.github.io/picard/ accessed on 13 May 2022] was used to mark PCR duplications. Differential analysis and annotation of differential sites (DERs) to associated genes and promoter regions were performed using diffReps (version 1.55.5) with padj < 0.001 and |FC| > 2 [62]. A circle plot of differential sites was generated using Circlize (version 0.3.10) [63]. GREAT (version 3.0.0) was used for prediction of associated GO terms with default extension settings (binominal *p*-value < 0.05) [64]. ChIP-seq coverage tracks normalized to 1 X sequencing depth and mean coverage plots around TSS (bin size = 50 bp) were generated with deepTools [65]. Coverage tracks were visualized in the IGV browser [66]. ATAC-seq data of male PHHs available on EGA (accession ID EGAS00001004201, n = 3) were processed as described for ChIP-seq data. ATAC peak calling was performed with MACS2 (https://github.com/taoliu/MACS, version 2.1.0 accessed on 13 May 2022). The overlap of the union of PHH ATAC peaks and histone modification DERs was calculated using GenomicRanges (v1.36.0, GenomicRanges, RRID:SCR_000025) [67]. 

### 2.10. Immunoblotting

Cells were lysed using a HEPES Buffer (50 mM HEPES pH7.5, 150 mM NaCl, 1 mM EDTA pH8, 2.5 mM EGTA pH 7.4, 0.1% Tween20, 10% glycerol, 0.1 mM orthovanadate Na, 1 mM NaF and 10 mM β-glycérophosphate). After cell lysis, 50 µg of total proteins were resolved on NuPAGE^®^ 4–12% Bis-Tris Gel electrophoresis (Invitrogen Waltham, MA, USA), transferred onto nitrocellulose membrane using iBlot system (Invitrogen). Chemiluminescence images were acquired with the Chemi-Capt software (Vilber Lourmat). Antibodies directed against CYP3A4 (1:800, Millipore Cat# 250308-100UL, RRID:AB_211764), CYP2E1 (1:1000, Oxford Biomedical Research Cat# PA26, RRID:AB_10816092), ALDOB (1:1000, Santa Cruz Biotechnology Cat# sc-12063, RRID:AB_2226680), CDK1 as previously described, MCM7 (1:1000, Santa Cruz Biotechnology Cat# sc-9966, RRID:AB_627235) cyclinA (1:1000, Santa Cruz Biotechnology Cat# sc-596 RRID:AB_631330)and HSC70 (1:1000; Santa Cruz Biotechnology Cat# sc-7298, RRID:AB_627761) were used [68]. 

### 2.11. Immunocytochemistry

Cells were fixed with 4% of paraformaldehyde (PFA). Immunolocalization of F-actin was performed with Alexa Fluor 594 Phalloidin (Thermo Fisher Scientific Cat# A12381, RRID:AB_2315633 Waltham, MA, USA). Cell nuclei were stained with Hoechst H33342 (B2261 Sigma) and images were acquired using the Cellomics ArrayScan VTI HCS Reader (Thermo Scientific). For extracellular matrix analysis, endogenous peroxidase activity was blocked and overnight incubation at 4 °C with antibodies against type III Collagen (Cell Sciences Cat# PS062, RRID:AB_2245094), type I Collagen (Cell Sciences Cat# PS060, RRID:AB_420256), type IV Collagen (Santa Cruz Biotechnology Cat# sc-59814, RRID:AB_1121796), Fibronectin (Santa Cruz Biotechnology Cat# sc-8422, RRID:AB_627598) or Laminin (Thermo Fisher Scientific Cat# PA1-16730, RRID:AB_2133633) was followed by incubation with a horseradish peroxidase-coupled secondary antibody (peroxidase-conjugated AffiniPure F(ab′)2 fragment anti-IgG (Jackson ImmunoResearch Labs Cat# 111-036-006, RRID:AB_2313586). Peroxidase staining was obtained with 3,3-diaminobenzidin/H2O2 solution. Cells were counterstained with Masson’s Hemalun and images were acquired using a Zeiss microscope.

### 2.12. Extracellular Matrix Deposition Staining

Reticulin staining by silver impregnation of extracellular matrix was performed as previously described [69].

### 2.13. DNA Synthesis

Percentages of proliferating HepaRG cells were estimated by the quantitation of cells that incorporated 5-Bromo-2′deoxyuridine (BrdU). HepaRG culture medium was supplemented with BrdU for 4 h and then cells were fixed with 4% of PFA. For staining, the primary antibody was an anti-BrdU (1:200, Abcam Cambridge, UK Cat# ab8152, RRID:AB_308713) and the secondary antibody was anti-mouse IgG-dylight488 (1:500, 072-03-18-18, KPL). Images were acquired using the Cellomics ArrayScan VTI HCS Reader. Pictures were analyzed with Cell Health Profiling BioApplication from Cellomics software.

### 2.14. Cytochrome P450 Activities

To determine CYP2A13 activity, a coumarin 7-hydroxylation assay was performed [70,71]. HepaRG cells were incubated for 2 h with 200 µM of coumarin (Ref C4261-Sigma Aldrich) in phenol red-free medium deprived of FCS and DMSO. To remove the conjugated form of 7-hydroxycoumarin, supernatants were incubated at 37°C for 2 h with glucuronidase/arylsulfatase (300 Fishman units/mL and 2400 Roy units/mL respectively). 7-hydroxylation of coumarin was then estimated by HPLC (Agilent 1100 series). 

### 2.15. Statistical Analysis

Results are expressed as means ± SD. Data were analyzed using Graph Pad Prism software (Version 4.0; GraphPad Prism, RRID:SCR_002798). The significance was evaluated by Student *t*-test for two-by-two comparison or by a one-way ANOVA followed by a post hoc Tukey for multiple comparisons.

## 3. Results

### 3.1. HepaRG Progenitors Spontaneously Differentiate into Biliary- and Hepatocyte-like Cells

To evaluate the gene expression changes during the differentiation of HepaRG from progenitors to mature hepatic cells and the role of DMSO during this process, we performed global gene expression analysis at various time points during HepaRG differentiation. We chose the progenitor stage, day 4 after cell seeding (D4), an intermediate stage, day fifteen (D15), which is committed in the differentiation and correspond to the time at which DMSO could be added for complete differentiation, the end of the differentiation period in the presence (D30 + DMSO) or absence of DMSO (D30 − DMSO), and finally, after DMSO removal, i.e., 30-day-old DMSO differentiated cells maintained for four additional days without DMSO (D34 ± DMSO) (Figure S1).

**Figure 1:**
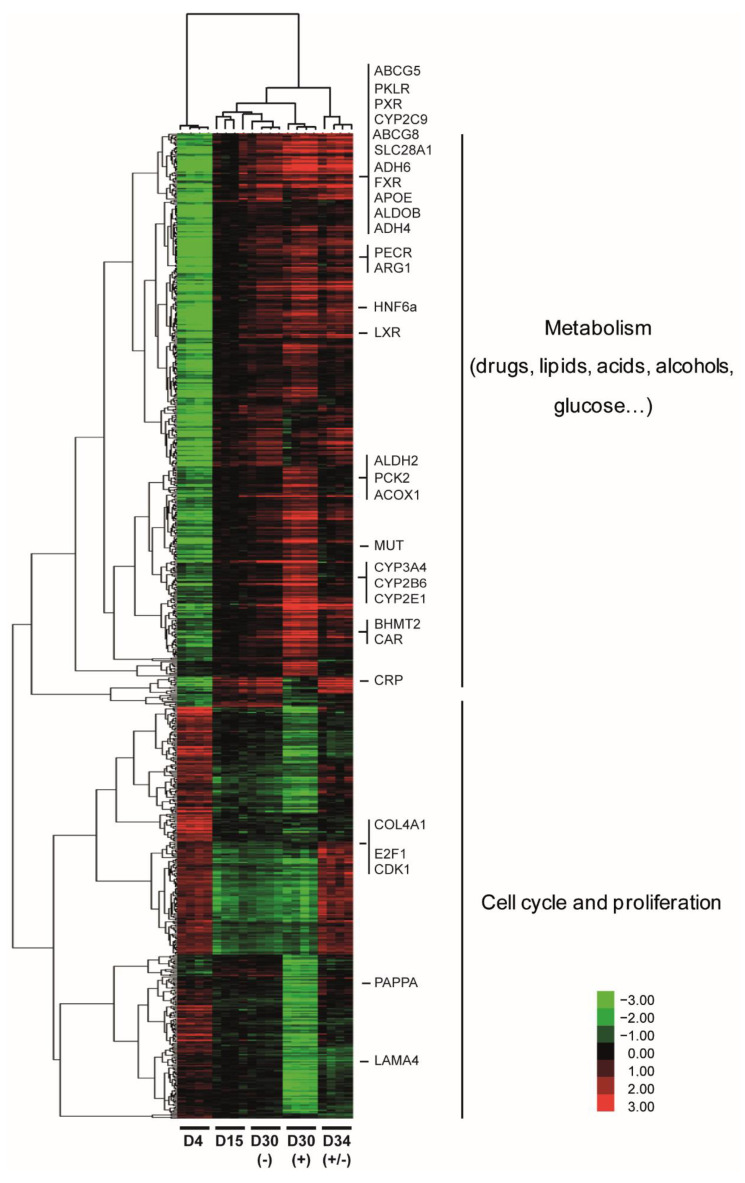
Global gene expression profile during HepaRG cell differentiation. Global gene expression data were obtained from HepaRG cells at the end of each culture condition (D4, D15, D30 − DMSO, D30 + DMSO, and D34 ± DMSO). Each culture condition was performed four times. Global gene expression data were filtered and the 775 significantly deregulated genes (*p* < 0.001, FC > 3) were hierarchically clustered with Gene Cluster 3.0. Clusters and dendrograms of experiments and genes were visualized by Treeview. Green indicates lower expression and red indicates higher expression. Representative biological functions of each cluster were evaluated using FuncAssociate 2.0 program with a significance cutoff of 0.005.

By a one-way ANOVA, we identified 775 deregulated genes (*p* < 0.001, FC > 3) in at least one condition during HepaRG differentiation. The hierarchical clustering of the differentially expressed genes revealed two main branches of the dendrogram associated with relevant biological functions (Figure 1). It separated the progenitor cells (D4) from the differentiating and differentiated cells (D15, D30 − DMSO, D30 + DMSO, and D34 ± DMSO). Interestingly, the most prominent transcriptional changes during the differentiation occurred between D4 and D15 highlighting that HepaRG differentiation itself occurred spontaneously without DMSO addition.

Dendrogram-grouped genes were annotated based on gene ontology enrichment determined by the FuncAssociate 2.0 program [56]. The upper part of the dendrogram included 451 genes whose expressions were upregulated at day 15 of the differentiation program. Among these upregulated genes major hepatic transcription factors are found such as the Liver X Nuclear Receptor (LXR, NR1H3), the Farnesoid X Receptor (FXR, NR1H4), the Constitutive Androstane Receptor (CAR, NR1I3) or the nuclear receptor Pregnane X receptors (PXR, NR1I2) (Figure S2). Indeed, the majority of the genes belonging to the first cluster were involved in the metabolism of (i) drugs, as shown by the presence of several cytochrome P450 family members (CYP3A4, CYP2B6, CYP2C9, or CYP2E1), (ii) lipids, as shown by the expressions of acyl-CoA oxidase 1 (ACOX1) and peroxisomal trans-2-enoyl-CoA reductase (PECR) that are involved in the beta-oxidation pathway and chain elongation of fatty acids, respectively, (iii) amino acids, with as example arginase (ARG1) that is involved in the urea cycle, (iv) alcohols, demonstrated by the presence of several members of the aldehyde dehydrogenase family (ALDH2, ADH4, ADH6…), and (v) glucose, as shown by the expressions of pyruvate kinase PKLR, which plays a key role in glycolysis, and the phosphoenolpyruvate carboxykinase 2 (PCK2), a key enzyme of the gluconeogenesis. Among these differentially expressed genes (DEGs), we also found genes expressed by biliary cells such as the transcription factors HNF6A and SOX9, the transporters ABCG5, ABCG8, and ABCA1, as well as the organic solute transporter SLC51B and SLC28A1 (Figure 1 and data not shown).

The lower part of the dendrogram clustered 324 genes whose expressions were mainly downregulated during HepaRG differentiation. These genes were mainly related to cell cycle progression and proliferation. This cluster included the cyclin-dependent kinase 1 (CDK1) and the E2F transcription factor 1 (E2F1), both essential for G1/S phase transition and which may regulate other genes in this cluster. Consistent with the role of basement membrane components on cell behaviors including proliferation, this cluster also grouped the genes relating to the extracellular region or collagen binding, such as laminin alpha 4 (LAMA4) and type IV collagen alpha 1 (COL4A1), two major constituents of the basement membrane [72]. These genes were highly expressed at the progenitor stage and then downregulated during the differentiation process.

### 3.2. Pleiotropic Effects of DMSO Enhance Several Hepatic Specific Functions in HepaRG Cells

**Figure 2:**
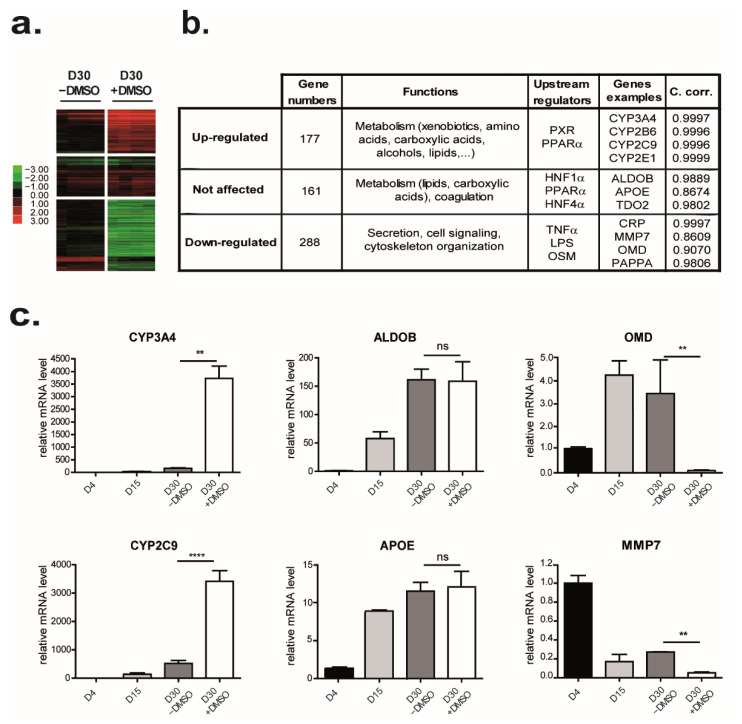
Effects of DMSO on HepaRG cell differentiation. Genes were grouped based on the effects of DMSO addition during the latter part of the HepaRG cell differentiation procedure. (**a**) Heat map of the 3 groups of genes: upregulated, not affected, and downregulated by DMSO exposure. Green indicates lower expression and red indicates higher expression. (**b**) Top functional categories and upstream transcriptional regulators associated with each gene group highlighted by IPA were summarized in the table. The coefficient of correlation (between microarray and real-time RT-PCR data) was calculated for each mentioned gene. (**c**) Genes whose expressions were upregulated (left panel), not affected (middle panel), and downregulated (right panel) were selected based on the microarray data. The expression of each selected gene was confirmed by real-time RT-PCR (n = 3). Results are expressed as relative to condition D4, arbitrary set to 1. ** *p* < 0.01; **** *p* < 0.001; ns: not significant.

Hierarchical clustering revealed that only groups of genes were sensitive to DMSO exposure. Expression of these genes started spontaneously from D4 and continued increasing along culture time even in the absence of DMSO (D30 − DMSO, Figure 1). The addition of DMSO to the culture medium at D15 enhances the upregulation of many genes upregulated along the differentiation program. To determine more specifically genes upregulated by DMSO exposure, a comparison two by two of D30 − DMSO and D30 + DMSO revealed that expressions of 465 genes (*p* < 0.005, FC > 2) were modulated by DMSO addition (Figure 2a) and among them, expression of 177 genes was highly upregulated by DMSO exposure (Figure 2a,b and Table S2). These genes were mainly involved in the metabolic function of the liver such as metabolisms of xenobiotics such as CYP3A4, CYP2B6, and CYP2E1, amino acids such as S-methylmethionine-homocysteine S-methyltransferase (BHMT2), glutaminase 2 (GLS2) and methionine adenosyltransferase 1A (MAT1A), carboxylic acids such as PCK2 and CYP2C9, alcohols such as catalase (CAT) 7-dehydrocholesterol reductase (DHCR7) and CYP3A4, and lipids such as acetyl-coa acyltransferase 1 (ACAA1), 2-hydroxyacyl-coa lyase 1 (HACL1) and solute carrier family 27 member 1 (SLC27A1). Ingenuity pathway analysis (IPA) indicated that their upstream regulators were PXR and the nuclear transcription factor peroxisome proliferator-activated receptor alpha (PPAR α, both involved in drug and lipid metabolisms (Figure S2). Among these upregulated genes, we validated by real-time RT-PCR the strong increase in several CYP family members as CYP3A4 and CYP2C9 expression levels by DMSO exposure (Figure 2c left panel and data not shown).

In addition to the genes described above, expressions of 161 genes, representing 20.7% of the genes modulated during the differentiation program (ANOVA *p* < 0.001; FC > 3), were not affected by the presence of DMSO (Table S3). These genes, involved in metabolism (mainly lipids such as apolipoprotein (APO) E and APOM and carboxylic acids such as tryptophan 2,3-dioxygenase (TDO2) and methylmalonyl CoA mutase (MUT)) as well as in coagulation (such as coagulation factor X (F10) and coagulation factor II (F2)), were regulated by both PPAR α and the hepatocyte nuclear factor (HNF) 1 α and HNF4 α which act in development and metabolic homeostasis of the organism (Figure 2b). Consistent with these observations, RNA levels of aldolase B (ALDOB), a glycolytic enzyme, and the APOE, involved in the catabolism of triglyceride-rich lipoprotein constituents, are not affected by DMSO exposure (Figure 2c middle panel).

### 3.3. DMSO Plays a Role in Matrix and Cytoskeleton Remodeling and Has Anti-Inflammatory Properties

**Figure 3:**
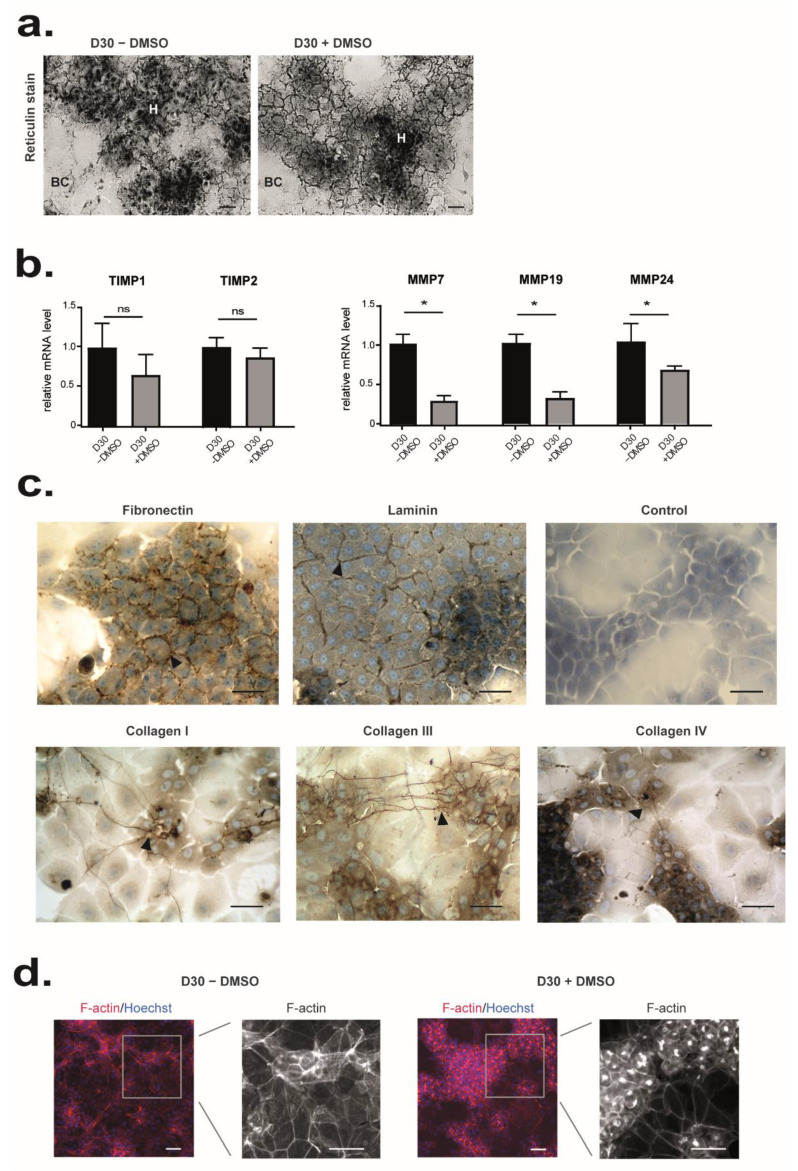
Effects of DMSO on matrix remodeling and cell organization. (**a**) ECM deposition was detected by reticulin staining on HepaRG cells differentiated from D15 of culture in the presence (D30+ DMSO) or the absence of DMSO (D30 − DMSO). (**b**) Microarray data for TIMP1, TIMP2, MMP7, MMP19, and MMP24 are expressed relative to conditions D30 − DMSO, arbitrary set to 1. The significance of DMSO treatment (D30 + DMSO vs D30 − DMSO) was evaluated by an unpaired *t*-test * *p* < 0.05, ns: not significant. (**c**) Immunostaining of fibronectin, laminin, collagen type I, III, and IV in HepaRG cells differentiated in the presence of DMSO (D30+ DMSO). Immunolocalization without primary antibody was performed as control. (**d**) Immunolocalization of F-actin in HepaRG cells differentiated from D15 of culture in the presence (D30+ DMSO, left panel) or in the absence of DMSO (D30 − DMSO, right panel). Nuclei were visualized by staining with Hoechst (shown in blue). Scale bars = 7 µm.

While DMSO has so far mainly been shown to induce genes involved in drug metabolism, we found that expression of 288 genes (62% of the modulated genes) associated with several gene ontology terms, such as secretion, cell signaling, or cytoskeletal organization, were significantly downregulated by DMSO exposure (*p* < 0.005, |FC| > 2) (Figure 2b and Table S4). For instance, mRNA levels of the metalloproteinase pregnancy-associated plasma protein A (PAPPA) that cleaves insulin-like growth factor binding proteins, the matrix metallopeptidase 7 (MMP7) involved in the breakdown of extracellular matrix, or the osteomodulin (OMD), a matrix organization related molecule, that suppresses the formation of collagen fibers, were decreased in the presence of DMSO (Figure 2c right panel). Some regulators of actin polymerization such as CDC42ep1 (CDC42 effector protein (Rho GTPase binding) (1), RHOBTB1 (Rho-related BTB domain containing 1), ABLIM2 (actin binding LIM protein family, member (2) or INF2 (inverted formin, FH2 and WH2 domain containing) were also found downregulated by DMSO. In agreement, extracellular matrix (ECM) deposition, evidenced by the reticulin staining, was barely detected in HepaRG from D30 − DMSO whereas it was clearly observed around the HepaRG-hepatocytes from D30 + DMSO (Figure 3a). In accordance, the ratio TIMP (tissue inhibitor of metalloproteinase 1)/MMP becomes favorable to ECM deposition in presence of DMSO (Figure 3b). This ECM was composed at least of fibronectin, laminin, and collagen I, III, and IV (Figure 3c). Some collagen fibers were also located between hepatocyte cords. In parallel, F-actin staining showed the presence of stress fiber in HepaRG cultured without DMSO whereas it revealed an F-actin mainly located at the cell periphery just beneath the plasma membrane in presence of DMSO (Figure 3d). An accumulation of F-actin around the bile canaliculus-like structures was also observed (Figure 3d).

The upstream regulator of these 288 genes identified by IPA suggested that DMSO had anti-inflammatory properties. Indeed, the tumor necrosis factor (TNF), a cytokine involved in the regulation of a wide spectrum of biological processes, the lipopolysaccharide (LPS), an endotoxin that induces an immune response, and the oncostatin M (OSM), a cytokine involved in growth regulation were identified to be involved in the regulation of these 288 repressed genes such as C-C Motif Chemokine Ligand 2 (CCL2), C-X-C Motif Chemokine Receptor 7 CXCR7 or C-Reactive Protein (CRP) (Figure 2b).

### 3.4. DMSO Induces Genome-Wide Histone Modification Patterns during HepaRG Differentiation

Unsupervised gene set enrichment analysis (GSEA) indicated a DMSO effect on chromatin remodeling. We identified enrichment (*p* < 0.05) of gene signatures associated with histone modifications as acetylation or methylation (Table S5). For instance, HepaRG cells differentiated with DMSO present an enrichment of the « PEART_HDAC_PROLIFERATION_CLUSTER_UP » corresponding to genes upregulated by histone deacetylase (HDAC) inhibitors [73]. We also highlighted the « WP_ETHANOL_EFFECTS_ON_HISTONE_MODIFICATIONS » corresponding to an altered histone modification pathway. To validate the role of DMSO on HepaRG differentiation, we analyzed the genome-wide patterns of H3K4me1, H3K4me3, and H3K27ac. These three canonical histone modifications are characteristic signatures for active promoters (H3K4me3) and enhancer elements (H3K4me1 and H3K27ac). We used ChIP-seq to generate and compare genome-wide profiles of these marks between D30 − DMSO and D30 + DMSO HepaRG cells. All experiments were performed in two independent replicates (Figure S3).

**Figure 4:**
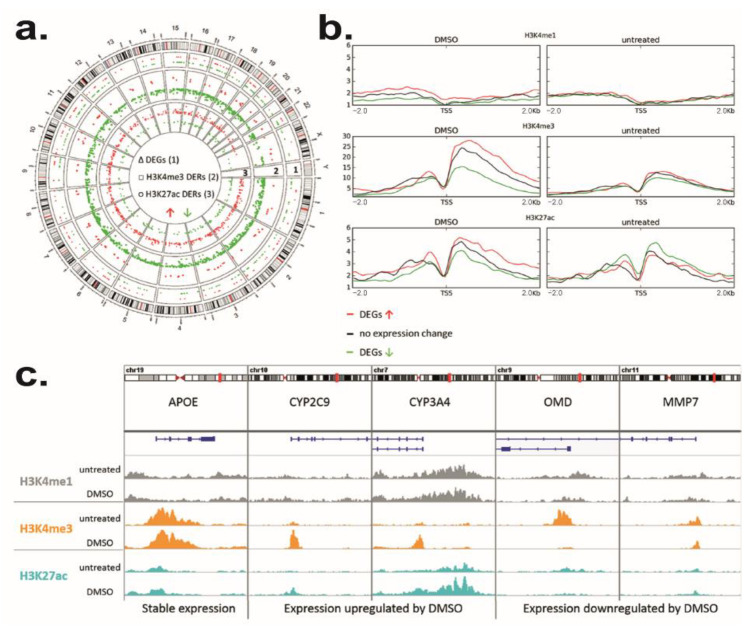
DMSO effect on genome-wide histone modification patterns in HepaRG differentiation. (**a**) Genome-wide distribution of DEGs (1, *p* < 0.005, FC> 2), H3K4me3 differentially enriched regions (DERs, 2) and H3K27ac DERs (3) upon DMSO exposure (padj< 0.001, FC > 2). Increase in expression or enrichment is colored in red, decrease in green. DERs are plotted as log2 fold changes ranging from −3 to 3. (**b**) Mean coverage of H3K4me1, H3K4me3, and H3K27ac HepaRG cells differentiated with and without DMSO (untreated) in a 2 kb window around TSS of genes with upregulated (red), downregulated (green) or unchanged (black) expression by cultivation with DMSO. (**c**) Exemplary histone modification patterns at unaffected (APOE), DMSO upregulated (CYP2C9, CYP3A4) and DMSO downregulated (OMD, MMP7) genes. H3K4me1 coverage is displayed in grey, H3K4me3 in orange, and H3K27ac in blue. Coverage tracks were normalized to their sequencing depth.

As a first analysis, we compared genome-wide changes using the program diffReps (padj < 0.001, FC > 2). This allows the identification of differentially enriched regions (DERs) and associated genes upon DMSO exposure. For H3K4me3 and H3K27ac, we found distinct changes in response to DMSO treatment (Figure 4a). For H3K4me1, we only observed a slight trend of a regional enrichment around transcriptional start sites (TSS) of upregulated genes (Figure 4b). Prominent DMSO-dependent loss of H3K4me3 was observed in 1140 DERs, while only 86 DERs gained H3K4me3 (Table S6). Moreover, 690 DERs showed an increase and 226 DERs a decrease in H3K27ac upon DMSO treatment (Table S7). 

The majority of H3K4me3 (76%) and H3K27ac (63%) DERs were found in gene context (Figure S4). 54% of H3K4me3 DERs were located in annotated promoter regions. H3K27ac DERs were more often detected at gene bodies (37%) or intergenic regions (37%). Interestingly, 73% of DERs with an increase in H3K27ac overlapped with accessible chromatin in PHHs that might correspond to regulatory elements relevant to hepatocyte cell identity. Genes with an upregulated expression showed a higher mean coverage of H3K4me3 and H3K27ac as compared to unchanged and especially downregulated genes in DMSO exposed HepaRG cells (Figure 4b). Indeed, 102 downregulated genes showed reduced H3K4me3 enrichment (141 DERs) with 8 genes also featuring reduced H3K27ac enrichment (Table S4). In 17 upregulated genes higher H3K4me3 (20 DERs) and in 25 genes increasing H3K27ac enrichment (136 DERs) was observed (Table S2). However, DERs did not necessarily coincide with differential gene expression. Seven H3K4me3 DERs and nineteen H3K27ac DERs were associated with genes not affected by DMSO exposure (Table S3) including liver-relevant genes such as ALDOB (Figure S4b). Moreover, a low number of DERs showed a negative correlation with gene expression. DERs with reduced H3K4me3 enrichment associated with upregulated genes (5) as UDP glucuronosyltransferase family 1 member A8 (UGT1A8) or H3K27ac DERs with increased enrichment located at downregulated genes (6) might represent alternative promoters of transcript variants or distal regulatory elements (Figure S4c).

The biological functions of upregulated DEGs associated with H3K4me3 and/or H3K27ac DERs such as CYP2C9 or CYP3A4 (Figure 4c) were mostly related to the metabolism of xenobiotics, lipids, amino acids, carboxylic acids, and alcohols. Genes that were not affected by DMSO, but nevertheless exhibited increasing H3K27ac enrichment was associated with liver-specific functions as well (Table S8). Stable genes without histone modification changes also included liver-related genes such as APOE (Figure 4c). Prominent examples of genes downregulated by DMSO with coinciding histone modification changes are OMD and MMP7 both involved in extracellular matrix remodeling (Figure 4c).

### 3.5. Identification of Molecules That Could Mimic or Reverse the DMSO Effect

**Figure 5:**
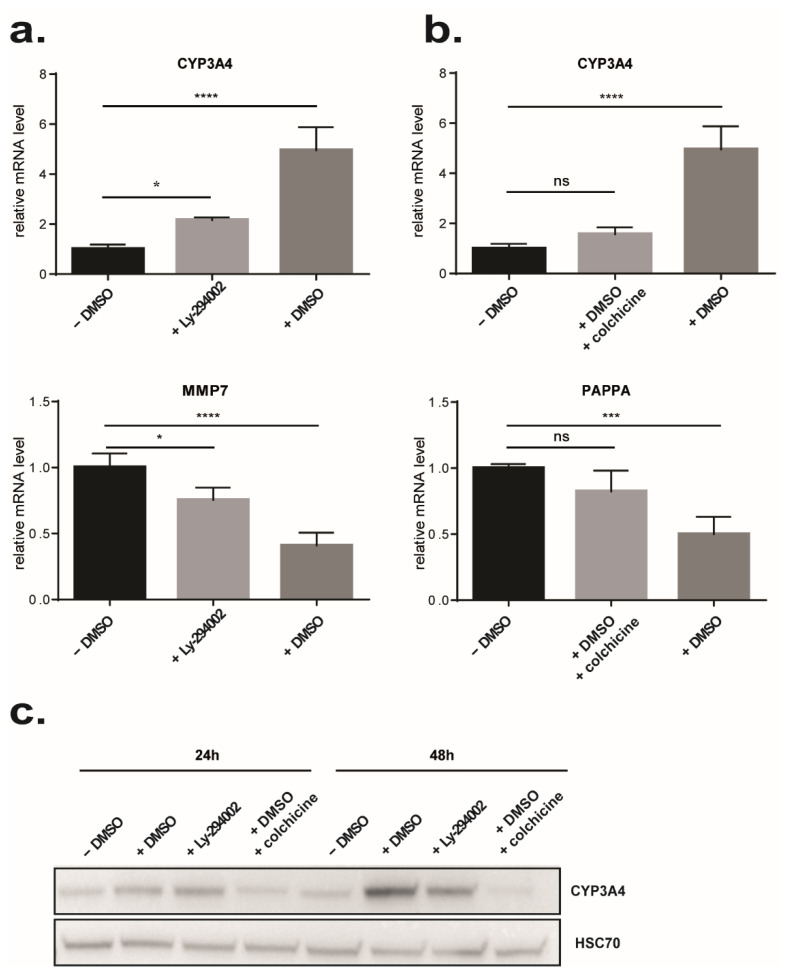
Comparison of DMSO effects with LY294002 and colchicine effects. At day 15 of differentiation HepaRG cells were treated for 24 h with 2% DMSO as positive control or with 10 µM LY294002, or 2% DMSO + 10 µM colchicine or let in classical medium without DMSO (negative control). (**a**) Effect of LY294002 treatment on CYP3A4 and MMP7 expressions was confirmed by real-time RT-PCR. (n = 4). (**b**) Effect of colchicine treatment on CYP3A4 and PAPPA expressions was confirmed by real-time RT-PCR. (n = 4). Results are expressed as relative to condition HepaRG D15 (24 h), arbitrary set to 1. * *p* < 0.05, *** *p* < 0.001, **** *p* < 0.0001. ns: not significant. (**c**) Effects of DMSO, Ly294002, and colchicine on the protein level of CYP3A4 were analyzed by Western blot after 24 h and 48 h of treatment.

To characterize further the effect of DMSO on gene expression, we used a connectivity map approach as previously described [59]. Among the top ten ranked molecules, we identified six molecules that could induce a similar signature to HepaRG cells treated by DMSO and four molecules presenting an inverse signature suggesting that they could reverse the global gene expression profile induced by DMSO exposure (Table S9). Among the molecules that could mimic DMSO effects, half are HDAC inhibitors: vorinostat, trichostatin A (TSA), and CP-690334-01, corroborating the signatures found previously and reinforcing the impact of DMSO on chromatin remodeling [74,75,76]. Of note, the development of the synthetic HDAC inhibitor vorinostat originated from DMSO [77]. Interestingly, we also identified molecules that have anti-inflammatory properties such as withaferin A and LY-294002. Regarding molecules that displayed inverse DMSO signature, we found molecules with various functions. Among them is colchicine, a well-known cytoskeletal drug that inhibits microtubule polymerization. Colchicine has also been shown to modulate extracellular matrix accumulation and to have anti-inflammatory properties [78,79]. To assess the role of DMSO, we selected 2 drugs: LY294002, a strong PI3K inhibitor, which may mimic the effect of DMSO, and colchicine, which rather may have an opposite effect. As expected, 24 h treatment of HepaRG-D15 with LY294002 led to an increase in CYP3A4 expression, a gene that was previously shown to be induced by DMSO, and a decrease in MMP7 expression, a gene downregulated by DMSO (Figure 5a–c). Conversely, 24 h treatment of HepaRG-D15 with 2% DMSO in the presence of colchicine showed that colchicine abolished the DMSO effect on CYP3A4 and PAPPA expressions (Figure 5b,c).

### 3.6. DMSO Removal Induces Transient Cell Proliferation

Since DMSO is removed at D30 from the culture media in some protocols of metabolism studies such as in CYP induction tests, we further compared the expression profiles of cells for which DMSO was removed from the culture media at day 30 for a 4-day period (D34 ± DMSO) with cells differentiated in the presence of DMSO (D30 + DMSO).

**Figure 6:**
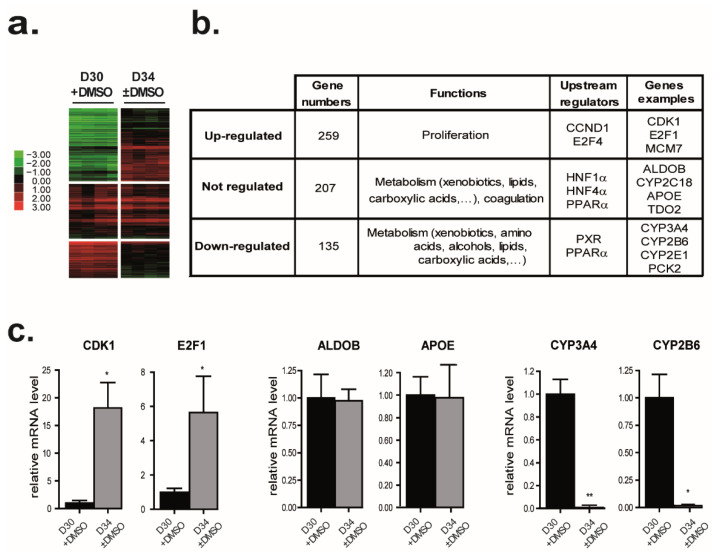
Effects of DMSO removal from the differentiated HepaRG cell culture on gene expressions. DMSO was removed from the HepaRG cell culture medium after the completion of hepatocyte differentiation at D30 + DMSO. Genes were grouped based on the effects of DMSO removal. (**a**) Heat map of the 3 groups of genes: downregulated, upregulated, and not affected by DMSO removal. Green indicates lower expression and red indicates higher expression. (**b**) Top functional categories and upstream transcriptional regulators associated with each gene group highlighted by IPA were summarized in the table. (**c**) Genes whose expressions were downregulated (upper panel), upregulated (middle panel), and not affected (lower panel) after the DMSO removal were selected based on the microarray data. The expression of each selected gene was confirmed by real-time RT-PCR. (n = 3). Results are expressed as relative to condition D30 + DMSO, arbitrary set to 1. * *p* < 0.05, ** *p* < 0.01.

Comparison two by two of D30 + DMSO and D34 ± DMSO revealed that expressions of 394 genes were modulated by DMSO removal (Figure 6a). Interestingly, only 37% (107) of the genes significantly repressed (*p* < 0.005; |FC| > 2) by the DMSO addition were also upregulated by the DMSO removal with the same stringency (Figure S5). While hierarchical clustering of deregulated genes in at least one condition during HepaRG differentiation (D4, D15, D30 − DMSO, D30 + DMSO, and D34 ± DMSO) revealed that cell cycle-related genes were strongly downregulated in differentiating cells (Figure 1), DMSO removal re-induced their expressions. Indeed, 259 genes mainly involved in cell cycle regulation and progression were significantly upregulated after DMSO removal (*p* < 0.005, FC > 2) (Figure 6b and Table S10). The upstream regulators of these genes are Cyclin D1 (CCND1) and E2F4 (Figure 6b). Among these genes, expressions of E2F1, a transcription factor controlling genes regulating S phase entry and DNA synthesis as well as CDK1, which is essential for G1/S and G2/M phase transitions of hepatocytes, were induced after DMSO removal (Figure 6c left panel) [80,81]. 

**Figure 7:**
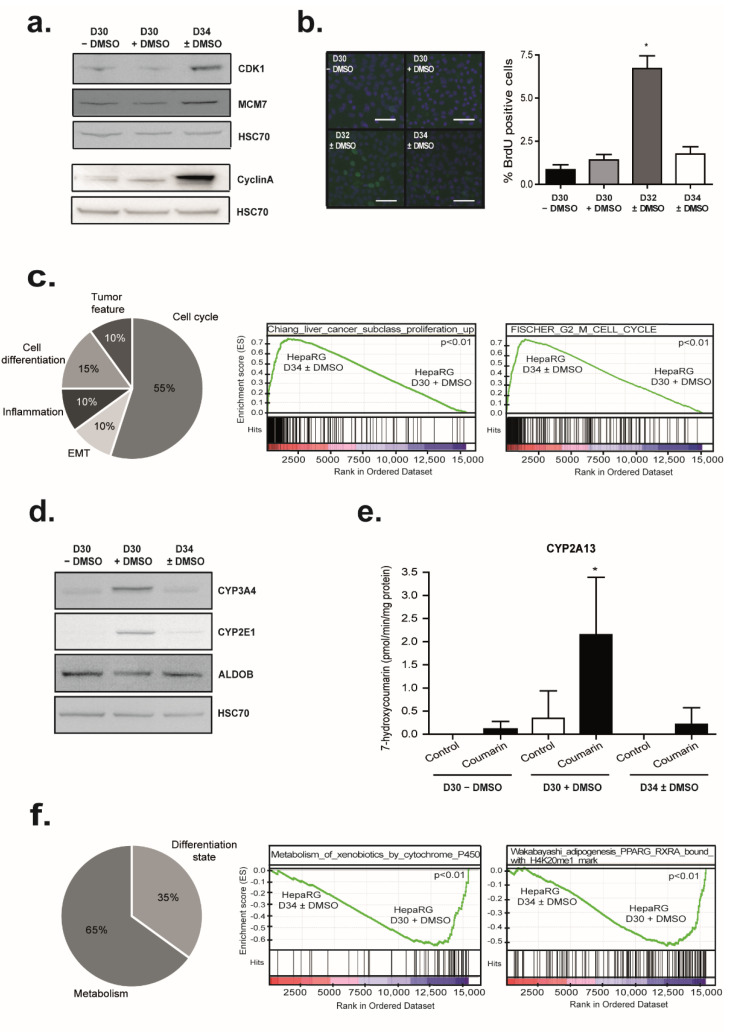
Transient cell cycle entry and modulation of metabolic functions of differentiated HepaRG cells after DMSO removal. (**a**) Effects of DMSO removal on the protein level of cell cycle-regulated genes (CDK1, MCM7, and CyclinA) were analyzed by Western blot (n = 3). HSC70 was measured as an internal control. (**b**) Transient induction of HepaRG cell proliferation after DMSO removal was analyzed by measuring BrdU incorporation (n = 3). Immunolocalization of HepaRG cells with anti-BrdU antibody. BrdU positive cells were shown in green, and nuclei were in blue (Hoechst). Scale bars = 50 µm. Quantification of BrdU incorporation was performed by Cell Health Profiling BioApplication from Cellomics software. * *p* < 0.05. (**c**) GSEA analysis using the gene expression profiles of HepaRG at D30 + DMSO and HepaRG at D34 ± DMSO. Functional repartition of the top 20 significant signatures enriched after DMSO removal. GSEA revealed an enrichment of the “Chiang_liver_cancer_subclass_proliferation_up” signature and the “Stein_ESR1_targets” signature. (**d**) Protein level of drug metabolism genes (CYP3A4, CYP2E1, and ALDOB) were analyzed by Western blot to confirm the gene expression changes after DMSO removal from the culture medium (n = 3). HSC70 was measured as an internal control. (**e**) Effect of DMSO removal on drug-metabolizing enzyme activities in HepaRG cells. HepaRG cells were incubated for 2 h with 200 µM of coumarin to measure CYP2A13 activity. 7-hydroxylation of coumarin was estimated by HPLC (n = 3). * *p* < 0.05. (**f**) GSEA analysis using the gene expression profiles of HepaRG at D30 + DMSO and HepaRG at D34 ± DMSO. Functional repartition of the top 20 significant signatures lost after DMSO removal. GSEA revealed an enrichment of the “Metabolism_of_xenobiotics_by_cytochromes_P450” and the “Wakabayashi_adipogenesis_PPARG_RXRA_bound_with_H4K20me1_mark” signature.

These observations were strengthened with the increase in CDK1 and minichromosome maintenance complex component 7 (MCM7) protein amounts after the DMSO removal, both essential for the initiation of eukaryotic genome replication (Figure 7a). Similarly, the expression of CyclinA (CCNA2) involved in both the S phase and the G2/M transition of the cell cycle was induced (FC = 7.3; *p*-value = 0.007) by DMSO removal (Figure 7a). However, the ability of HepaRG cells to replicate DNA after DMSO removal was low and transient (Figure 7b). While 1.5 to 2% of differentiated HepaRG cells, mainly hepatocytes, were BrdU positive in both D30 − DMSO or D30 + DMSO conditions, 7% of differentiated HepaRG cells became BrdU positive two days after the DMSO removal (Figure 7b). This percentage returned to the basal level four days later. Consistent with these observations, unsupervised GSEA identified significant enrichment of several signatures mainly related to cell proliferation (55% of the top 20 significant signatures), cell differentiation, epithelial to mesenchymal transition (EMT), inflammation, or tumor feature. Among them, the “CHIANG_LIVER_CANCER_SUBCLASS_PROLIFERATION_UP” described by Chiang et al. and the “FISCHER_G2_M_CELL_CYCLE” described by Fisher et al. [82,83] (Figure 7c).

### 3.7. DMSO Removal Differentially Modulates Some Metabolic Functions

Among the 394 genes modulated by the DMSO removal (D34 ± DMSO versus D30 + DMSO), 135 genes were significantly downregulated (*p* < 0.005, |FC| > 2) (Table S11). Of note, 58.5% (79) of these 135 genes were found to be significantly induced by DMSO with the same stringency (Figure S5). These genes were mainly involved in metabolisms (xenobiotics, amino acids, alcohols, lipids, organic acids…) and IPA reported that upstream regulators of these genes are PXR and PPAR α. Among these downregulated genes, we identified several cytochrome P450 family members such as CYP3A4, CYP2B6, CYP2E1, or CYP2A13 (Figure 6b). As previously described in other studies, we confirmed that mRNA levels of CYP2E1 and CYP3A4 were strongly decreased 4 days after DMSO removal (Figure 6c middle panel and Figure 7d) [18,22]. Accordingly, a reduction in CYP3A4 activity after DMSO removal was detected (Figure S6). As well, CYP2A13 was identified as downregulated after DMSO removal in our global gene expression data. Consistent with this observation, CYP2A13 activity, measured by detecting the 7-hydroxylation of coumarin, was increased in HepaRG cells differentiated in the presence of DMSO in comparison to those differentiated in the absence of DMSO and as expected, its activity was strongly decreased after DMSO removal (Figure 7e).

Finally, unsupervised GSEA also identified a significant loss of several signatures mainly related to metabolism (65% of the top 20 significant signatures) or the differentiation state (35%). Among them are the KEGG pathway “KEGG_METABOLISM_OF_XENOBIOTICS_BY_CYTOCHROME_P450” and the “Wakabayashi_adipogenesis_PPARG_RXRA_bound_with_H4K20me1_mark” described by Wakabayashi et al. were observed in HepaRG cells after DMSO removal (Figure 7f) [84]. Interestingly, our comparison also highlighted that the expression of 26,7% of the genes modulated during the differentiation program (ANOVA *p* < 0.001; |FC| > 3), was not affected by the DMSO removal (Table S12 and Figure 6b). Such genes were also involved in metabolisms (xenobiotics, lipids, organic acids) and coagulation. Some cytochrome P450 family members such as CYP2C18 and other genes such as ALDOB and APOE were found in this gene group (Figure 6c right panel and Figure 7d). Their upstream regulators according to the IPA prediction were PPARα, HNF1α, and HNF4α (Figure 6b and Figure S2). Of note, 41% (95) of these genes were not found regulated by DMSO addition (Figure S5). Among them, are the transcription factor FXR, the circulating lipoprotein APOE, and APOH, involved in lipoprotein metabolism and coagulation, the Acetyl-CoA Acyltransferase 2 (ACAA1), the Paraoxonase (PON1) a secreted enzyme sharing ester hydrolase activity, or F2. Therefore, DMSO removal modulates only some genes involved in various metabolisms.

## 4. Discussion

DMSO is one of the most common solvents used in biological studies and was the first chemical used to induce growth arrest and terminal differentiation [85]. DMSO appears to alter the secondary structure of proteins as well as the structure of nucleic acid [86]. However, the molecular mechanisms by which DMSO influences cellular processes are not well understood and its role seems to be dependent on the cell type [87,88]. Regarding hepatocyte differentiation, DMSO is usually used at 2%. At this concentration, it has been shown to maintain the differentiated phenotype of PHHs [28] and to improve the maturation of HepaRG-hepatocyte-like cells which express hepatic markers at comparable levels to those expressed by PHHs [7,47]. In adult rat hepatocytes, DMSO could play as a hydroxyl radical scavenger [89], stabilizing the CYP3A4 protein levels [90] while it modulates lipid metabolism in human hepatoblastoma HepG2 cells [37,91]. In neonatal rat hepatocytes, DMSO reduces epithelial to mesenchymal transition [92]. Few studies describe the effect of DMSO on HepaRG drug metabolism [18,22,51]. In view of the multitude of effects of DMSO, we performed a transcriptomic and genome-wide ChIP-seq analysis of HepaRG cells to best characterize its molecular targets. We found that hepatic progenitor differentiation is characterized by extensive changes in mRNA levels and that DMSO induces hepatocyte maturation through mRNA regulation and widespread changes in histone modification. In addition to an undoubted effect on drug metabolism, we identified that DMSO treatment induced a global impact on cellular physiological processes in differentiated HepaRG cells. Alongside anti-inflammatory properties, DMSO induces a lessening of PI3K signaling as well as cytoskeleton organization and ECM remodeling. Notably, the DMSO effect could be partly mimicked by Ly-294002, a PI3K inhibitor, or inhibited by colchicine, a microtubule-disrupting agent.

Colchicine and PI3K signaling are known to modulate cytoskeletal functions, such as cell polarity, intracellular compartmentalization, cell movement and division [93] as well as ECM remodeling. For example, activation of the PI3K-signaling pathway induces MMP2 expression in response to cytoskeletal destabilization in endothelial cells during angiogenesis whereas inhibition of PI3K by Ly-294002 reduces MMP7 expression in triple-negative breast cancer MDA-MB468 cells [94] and colorectal cancer cells [95]. These inhibitions were associated with reduced proliferation and migration. Accordingly, MMP7 is downregulated by DMSO in HepaRG cells. Particularly, PI3K activation was recently shown to increase glycolysis fluxes by remodeling the actin cytoskeleton and mobilization of aldolase from F-actin through Rac activation in mammary epithelial cells [96]. Conversely, the PI3K inhibitor reduces glycolysis. This observation is coherent with the fact that glycolysis is mitigated in favor of oxidative phosphorylation and fatty acid oxidation in adult hepatocytes. As regards colchicine, it has anti-inflammatory properties, blocks the dynamic of microtubules, and modulates the expression of MMP and TIMP contributing to ECM remodeling [97]. In the liver, colchicine decreases the deposition of ECM and the presence of inflammatory molecules while it increases the expression of hepatocyte proliferation markers [98,99]. In addition, it was previously shown that colchicine by altering the microtubules network, downregulates the expression and the protein levels of the genes controlled by the glucocorticoid receptor such as CYP2B6, CYP2C8, CYP2C9, CYP3A4, and CYP1A2 [100,101,102,103]. The importance of cytoskeleton-related genes in the expression and induction of CYP and drug transporter genes was also emphasized by the similarity between the HepG2 gene profiles in 2D cultures treated with docetaxel, a tubulin-stabilizing agent, and in 3D cultures, which enhances the expression of a variety of drug-metabolism related genes and several microtubule components [104]. Importantly, it was more recently shown that the cytoskeletal networks (actin microfilaments, microtubules, and keratin intermediate filaments 8/18) were involved in the proper interplay between insulin receptor, glucose transporter, and mitochondria in hepatocytes [105]. Likewise, integrin-focal adhesion kinase signaling and E-cadherin mechanotransduction were shown to modulate the epithelial cell metabolism and mitochondrial function through activation of STAT3 and AMP-activated kinase (AMPK), respectively [106,107]. Thus, DMSO through its impact on cytoskeleton organization and ECM remodeling, both processes previously described to coordinate the regulation of hepatic specific gene expression, could favor the maturation of the HepaRG-hepatocytes [69]. Altogether, these observations also emphasize the link between cell shape, metabolism, and differentiation.

This broad impact on the cellular physiological process leading to phenotypic changes is associated with post-translational modifications on the N-termini of histones, known to play a critical role in the epigenetic mechanism for gene regulation [108,109]. DMSO induced a distinct effect on the active histone modifications H3K4me3 and H3K27ac during HepaRG differentiation partially coinciding with gene expression changes. These results are similar to findings obtained by TSA treatment on liver cell lines and PHHs, demonstrating a good correlation between mRNA expression and histone modification [110]. While HepaRG cells differentiated without DMSO showed gene signatures associated with HDACs and genes with H3K4me3 marks in promoters, HepaRG cells differentiated in presence of DMSO presented an enrichment for genes regulated by histone acetylation. The majority of H3K27ac DERs, characteristics of active regions, showed increased enrichment in response to DMSO exposure, while H3K4me3 DERs featured mostly reduced enrichment. Hence, the long-term DMSO exposure has a significant effect on the promoter and enhancer-specific histone acetylation accompanied by an influence mainly on promoter H3K4me3. Some histone H4 arginine 3 methylation has been identified in RARA targeted promoters in HL60 cells primed with DMSO for 16–24 h [111]. Recently, histone modification (methylation and acetylation) has been described in the regulatory region of PXR in the HepG2 hepatoblastoma cells stably transfected with 3xFlag-PXR and treated for 18 h with DMSO [46]. This modification was dose-dependent and transient since 72 h after DMSO removal the primed and naïve cells had no difference in PXR target gene induction. In our HepaRG model, we observed that the 15 days of exposure to DMSO repressed MMP7 with a high correlation with histone modification pattern. It also induced histone modifications for various nuclear receptors involved in drug-metabolizing enzyme regulation. H3K27ac in promoter was increased for PXR and H3K4me3 in promoter was augmented for CAR. Regarding AHR, we observed an increase in H3K27ac in the promoter and a decrease in H3K4me3 in the genebody. Concerning, PPARs, H3K27ac was increased in PPARG genebody whereas no histone modification enrichment was detected for PPARD and PPARA despite they were induced as well as their target genes by DMSO (Figure 2 and Figure S2). Altogether, our results showed that DMSO leads to increased drug and lipid metabolisms, the regulation of cholesterol biosynthesis as well as induction in amino acid, vitamin and co-factor, and steroid metabolisms. However, it is interesting to note that no significant changes were observed for HNF4α and HNF1 α which contribute to liver development and expression of several hepatic genes as well as LXR and FXR that function as intracellular sensors for sterols and bile acids, respectively (Figure S2). Their expressions were indeed rapidly induced during the HepaRG differentiation program and before the DMSO addition. More specifically, the spontaneous expression of HNF4 α, which precedes maturation of differentiating HepaRG cells resulted from a shift in 5 hmC at the HNF4 α locus which occurs during the first week of cell culture [112]. Thus, these results showed the sequential expression of nuclear receptors along the hepatic differentiation program and highlighted those that trigger hepatocyte maturation. 

In addition to the nuclear receptor modifications, we also observed that the expression of various metabolizing enzymes was correlated with an increase in H3K4me3 marks (CYP2E1, CYP2B6, CYP2C19, CYP4F2) or H3K27ac marks (CYP2C9, CYP3A5, UGT2B15, UGT1A6, UGT1A8) whereas CYP3A4 showed both H3K4me3 and H3K27ac marks. Importantly, we detected that the increase in H3K27ac marks in the genebody of the cytochrome P450 oxidoreductase (POR) is associated with its higher expression. This is particularly interesting regarding the essential role of this enzyme in the proper functioning of the CYP. Indeed, by donating electrons directly from NADPH to all microsomal P450 enzymes, POR allows the oxidation of their substrates. Regarding ECM organization, among the downregulated genes, we found OMD, MMP7, and 19, which display a decrease in H3K4me3. Interestingly, OMD is involved in an extracellular matrix organization and suppresses the formation of collagen fibers [113] while MMP19 and MMP7 hydrolyze ECM components such as collagen type IV, laminin, nidogen, and fibronectin [114,115]. Therefore, their downregulation upon DMSO treatment favors matrix organization and deposition by slowing the rate of basement membrane destruction and ECM remodeling.

The DMSO effect was partly reversible since only part of the genes repressed by DMSO exposure were upregulated by DMSO removal. Interestingly, DMSO removal induces new target genes mainly involved in cell proliferation although the proliferation rate stayed low (7%). These data fit with previous studies reporting that DMSO decreases cell proliferation and cytokine production in human peripheral blood mononuclear cells (PBMC) [116]. In the same way, just under half of the genes induced by DMSO exposure were downregulated by DMSO removal. These genes were mainly under the control of upstream regulators, such as PXR, regulated by DMSO exposure. The genes with unchanged expression were mainly under the control of transcriptional factors such as HNF4 α, HNF1 α, insensitive to DMSO exposure, and PPAR γ, the expression of which remained stable after DMSO removal.

## 5. Conclusions

In conclusion, our results showed that DMSO favors hepatocyte maturation partly through post-translational modifications on the N-termini of histones. Our study also highlighted the sequential expression of nuclear receptors, which punctuate the differentiation program of hepatic progenitors toward mature hepatocytes and demonstrated the interplay between the cytoskeleton and EMC remodeling with hepatocyte maturation and metabolism, altogether leading to an increase in our understanding of hepatocyte differentiation/maturation and the proper use of HepaRG cells in toxicology studies. 

## Data Availability

The data discussed in this publication have been deposited in NCBI’s Gene Expression Omnibus (GEO, RRID:SCR_005012). Transcriptomic data are accessible through GEO Series accession number GSE112123 since 25 July 2022 (https://www.ncbi.nlm.nih.gov/geo/query/acc.cgi?acc=GSE112123). The Chip-seq data are accessible through GEO Series accession number GSE179988 since 25 July 2022 (https://www.ncbi.nlm.nih.gov/geo/query/acc.cgi?acc=GSE179988).

## Supplementary Materials

The following supporting information can be downloaded at: https://www.mdpi.com/article/10.3390/cells11152298/s1, Figure S1: HepaRG cell differentiation procedures; Figure S2: Expression of key transcription factors during HepaRG differentiation process; Figure S3: Quality of ChIP-seq data; Figure S4: Histone modification results; Figure S5: Impact of DMSO addition and DMSO removal on gene expression; Figure S6: Effect of DMSO removal on drug-metabolizing enzyme activities in HepaRG cells; Table S1: Primer sequences used for real-time RT-PCR; Table S2: List of significantly upregulated genes by DMSO exposure (*p* < 0.005, FC > 2); Table S3: List of not affected genes by DMSO exposure (*p* < 0.005, FC > 2); Table S4: List of significantly downregulated genes by DMSO exposure (*p* < 0.005, FC > 2); Table S5: GSEA using the whole C2 collection of MsigDB; Table S6: Genomic localization of H3K4me3 DERs including adjusted *p*-values (padj), fold changes (log2FC), associated genes (GName) and genomic features (Feature); Table S7: Genomic localization of H3K27ac (B) DERs including adjusted *p*-values (padj), fold changes (log2FC), associated genes (GName) and genomic features (Feature); Table S8: Top 100 gene ontology biological process terms; Table S9: Connectivity Map (Cmap) results; Table S10: List of significantly upregulated genes after DMSO removal (*p* < 0.005, FC > 2); Table S11: List of significantly downregulated genes after DMSO removal (*p* < 0.005, FC > 2); Table S12: List of not affected genes after DMSO removal.
