# Supplementary material for: Transcriptional and Epigenetic Consequences of DMSO Treatment on HepaRG Cells
Source: Cells. 2022 Jul 26;11(15):2298. doi: 10.3390/cells11152298 (PMC9331440; doi:10.3390/cells11152298)
Supplement: Supplementary file 1 [file cells-11-02298-s001.zip › Supplementary informations.pdf]

Supplementary Materials

For the detection of CYP3A4 activity, HepaRG cells were incubated 2h with 200 µM of testosterone (Ref T1500-Sigma Aldrich) in phenol red-free medium deprived in FCS and DMSO. 6 -hydroxylation of testosterone was then quantified by HPLC [19]. For studying the inducibility of the CYP3A4, HepaRG cells were treated for 24h with 50 µM of rifam-picin (Ref R3501-Sigma-Aldrich) before the incubation with testosterone.

Supplementary Figures

Figure S1. HepaRG cell differentiation procedures. (a) Culture conditions of HepaRG cell applied in this study. (b) HepaRG cell morphology at the end of each cell culture condition. Scale bars = 50 µm. White arrow: medium not supplemented with 2% DMSO. Black arrow: medium supplemented with 2% of DMSO.

Figure S2. Expression of key transcription factors during HepaRG differentiation process. Microarray data are expressed as relative to condition D4, arbitrary set to 1. (a) Genes whose expressions were significantly regulated along differentiation process (compared to D4, ANOVA $p<0.05$). (b) Gene not significantly regulated along differentiation process (compared to D4, ANOVA $p<0.05$). The significance of DMSO treatment (D30+DMSO vs D30-DMSO) was evaluated by an unpaired t-test * $p<0.05$, ** $p<0.01$, *** $p<0.005$, **** $p<0.001$, ns: no significant.

Figure S3. Quality of ChIP-seq data. (a) Number of mapped reads per sample. (b) Pearson correlation of H3K4me1, H3K4me3 and H3K27ac in promoter regions.

Figure S4. Histone modification results. (a) Annotation of H3K4me3 and H3K27ac differentially enriched regions to their genomic context (promoter, gene body and intergenic). (b) Histone modification patterns at the ALDOB gene with stable expression but differential H3K4me3 and H3K27ac enrichment upon DMSO treatment. H3K4me1 coverage is displayed in grey, H3K4me3 in orange and H3K27ac in blue. Coverage tracks were normalized to their sequencing depth. (c) Exemplary histone modification patterns of DERs with reduced enrichment localized in up-regulated DEGs upon DMSO treatment. The expression of UGT1A6 and UGT1A8 is up-regulated. The H3K4me3 DER with reduced enrichment (red box) marks a possible alternative promoter of two transcript variants (UGT1A6 and UGT1A7).

Figure S5. Impact of DMSO addition and DMSO removal on gene expression. Venn diagram comparing the 6 datasets: genes affected by DMSO addition (up- or down-regulated), genes affected by DMSO removal (up- or down-regulated) and genes not affected by DMSO (addition or removal).

Figure S6. Effect of DMSO removal on drug-metabolizing enzyme activities in HepaRG cells. For CYP3A4 induction HepaRG cells were treated for 24h with 50 µM of rifampicin before the incubation with testosterone. 6β-hydroxylation of testosterone was quantified by HPLC. Here is shown one representative experiment.

Supplementary Tables

Table S1. Primer sequences used for real-time RT-PCR

Table S2. List of significantly up-regulated genes by DMSO exposure (p<0.005, FC>2). FC: Expression Fold Change. Red: increase of associated H3K4me3 or H3K27ac enrichment, green: decrease, grey: no significant change.

Table S3. List of not affected genes by DMSO exposure (p<0.005, FC>2). FC: Expression Fold Change. Red: increase of associated H3K4me3 or H3K27ac enrichment, green: decrease, grey: no significant change.

Table S4. List of significantly down-regulated genes by DMSO exposure (p<0.005, FC>2). FC: Expression Fold Change. Red: increase of associated H3K4me3 or H3K27ac enrichment, green: decrease, grey: no significant change.

Table S5. **GSEA using the whole C2 collection of MsigDB.** Genesets significantly enriched (p<0.05 and FDR < 0.25) in HepaRG cultured with DMSO (D30+DMSO vs D30-DMSO) (A) and in HepaRG cultured in absence of DMSO (D30-DMSO vs D30+DMSO) (B). MsigDB signatures can be found at www.broadinstitute.org/gsea/msigdb . NES: normalized enrichment score.

Table S6. Genomic localization of H3K4me3 DERs including adjusted p-values (padj), fold changes (log2FC), associated genes (GName) and genomic features (Feature).

Table S7. Genomic localization of H3K27ac (B) DERs including adjusted p-values (padj), fold changes (log2FC), associated genes (GName) and genomic features (Feature).

Table S8. Top 100 gene ontology biological process terms. Top 100 gene ontology biological process terms of up-regulated genes with increasing H3K4me3 enrichment, up-regulated genes with increasing H3K27ac enrichment and unchanged genes with increasing H3K27ac enrichment. GO terms found in more than one list are marked in yellow.

Table S9. Connectivity Map (Cmap) results. Connectivity Map (Cmap) results of comparison of HepaRG cultured with DMSO (D30+DMSO) versus HepaRG cultured without DMSO (D30-DMSO) expression.

Table S10. List of significantly up-regulated genes after DMSO removal (p<0.005, FC>2). FC: Fold Change.

Table S11. List of significantly down-regulated genes after DMSO removal (p<0.005, FC>2). FC: Fold Change.

Table S12. List of not affected genes after DMSO removal. FC: Fold Change.
