# Supplementary material for: Transcriptional and Epigenetic Consequences of DMSO Treatment on HepaRG Cells
Source: Cells. 2022 Jul 26;11(15):2298. doi: 10.3390/cells11152298 (PMC9331440; doi:10.3390/cells11152298)
Supplement: Supplementary file 1 [file cells-11-02298-s001.zip › TableS1.pdf]

**Genes**	**Forward Primers**	**Reverse Primers**
ALDOB	GCATCTGTCAGCAGAATGGA	TAGACAGCAGCCAGGACCTT
APOE	TTGTTCCTCCAGTTCCGATT	CTGCTCAGCTCCCAGGTC
CDK1	ACAAAGGAACAATTAAACTGGCTG	CTGGAGTTGAGTAACGAGCTG
CRP	GAACTTTCAGCCGAATACATCTTTT	CCTTCCTCGACATGTCTGTCT
CYP2B6	TTCCTACTGCTTCCGTCTATCAAA	GTGCAGAATCCCACAGCTCA
CYP2C9	GGACAGAGACGACAAGCACA	AATGGACATGAACAACCCTCA
CYP2E1	TTGAAGCCTCTCGTTGACCC	CGTGGTGGGATACAGCCAA
CYP3A4	CTTCATCCAATGGACTGCATAAAT	TCCCAAGTATAACACTCTACACAGACAA
E2F1	TGGACAAGGCACCCCCACCA	GGCCAGGCCAAAGTCAGCCA
MMP7	GAACGCTGGACGGATGGTA	CAAGTTCATGAGTTGCAGCATACA
OMD	ATCCCCCTAGCTCACCACAT	GCCTTGGGTCCTTAGTACCTG
PAPPA	TGCACTTAGGTGGCATTGTC	TGGGTTGGTATCATTGCAGA
TBP	GAGCTGTGATGTGAAGTTTCC	TCTGGGTTTGATCATTCTGTAG
