# Supplementary material for: Transcriptional and Epigenetic Consequences of DMSO Treatment on HepaRG Cells
Source: Cells. 2022 Jul 26;11(15):2298. doi: 10.3390/cells11152298 (PMC9331440; doi:10.3390/cells11152298)
Supplement: Supplementary file 1 [file cells-11-02298-s001.zip › TableS9.pdf]

| rank | cmap name | mean | n | enrichment | p | specificity |
|---|---|---|---|---|---|---|
| 1 | vorinostat | 0.584 | 12 | 0.830 | 0.00000 | 0.1005 |
| 2 | trichostatin A | 0.337 | 182 | 0.750 | 0.00000 | 0.0426 |
| 3 | LY-294002 | 0.248 | 61 | 0.408 | 0.00000 | 0.1342 |
| 4 | withaferin A | 0.470 | 4 | 0.926 | 0.00004 | 0.0474 |
| 5 | nefopam | -0.582 | 5 | -0.817 | 0.00052 | 0.0000 |
| 6 | colchicine | -0.501 | 6 | -0.733 | 0.00072 | 0.0000 |
| 7 | CP-690334-01 | 0.459 | 8 | 0.644 | 0.00094 | 0.0485 |
| 8 | imidurea | -0.563 | 3 | -0.917 | 0.00096 | 0.0000 |
| 9 | praziquantel | -0.489 | 4 | -0.840 | 0.00117 | 0.0000 |
| 10 | AG-013608 | 0.194 | 8 | 0.589 | 0.00345 | 0.0213 |
