# Supplementary figures and images for: Transcriptional and Epigenetic Consequences of DMSO Treatment on HepaRG Cells
Source: Cells. 2022 Jul 26;11(15):2298. doi: 10.3390/cells11152298 (PMC9331440; doi:10.3390/cells11152298)

a.

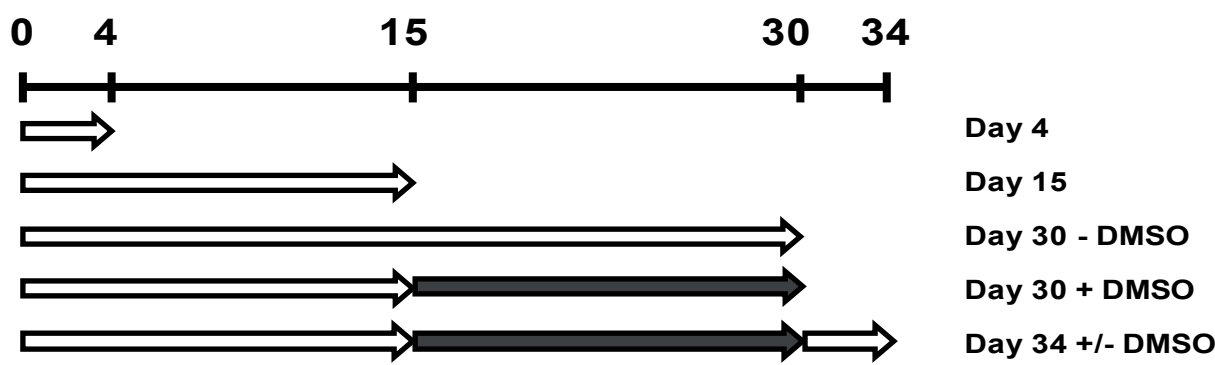

b.

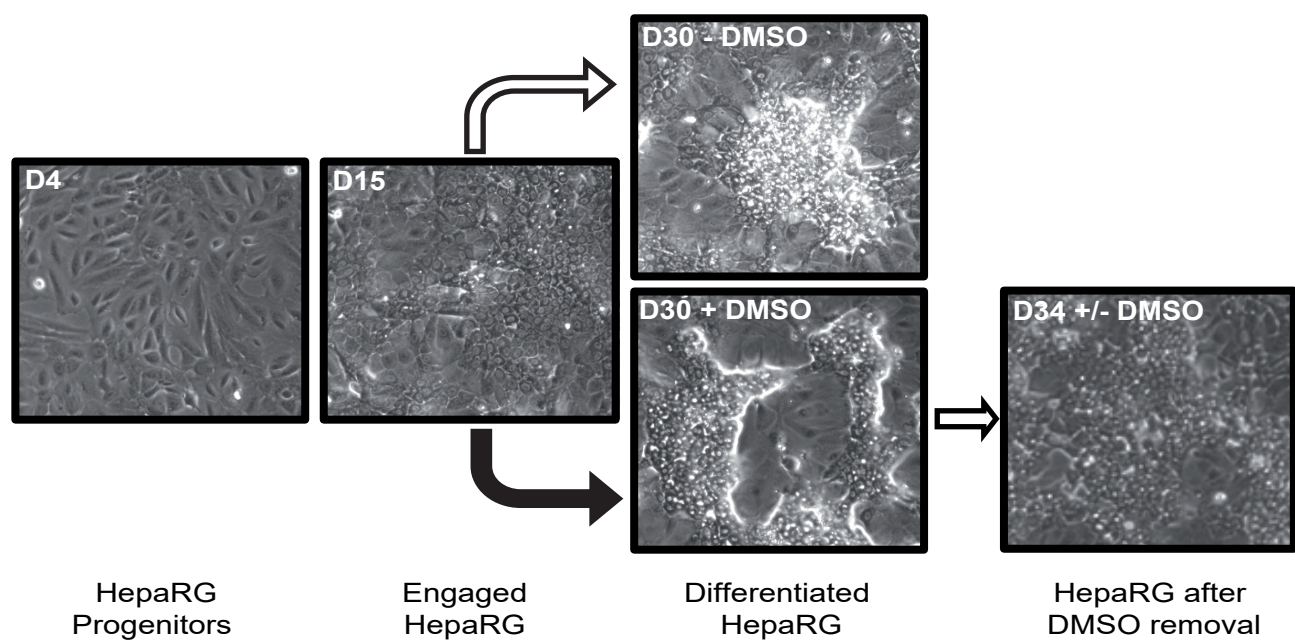

Figure S1

Supplement: Supplementary file 1 [file cells-11-02298-s001.zip › FigureS1.pdf]

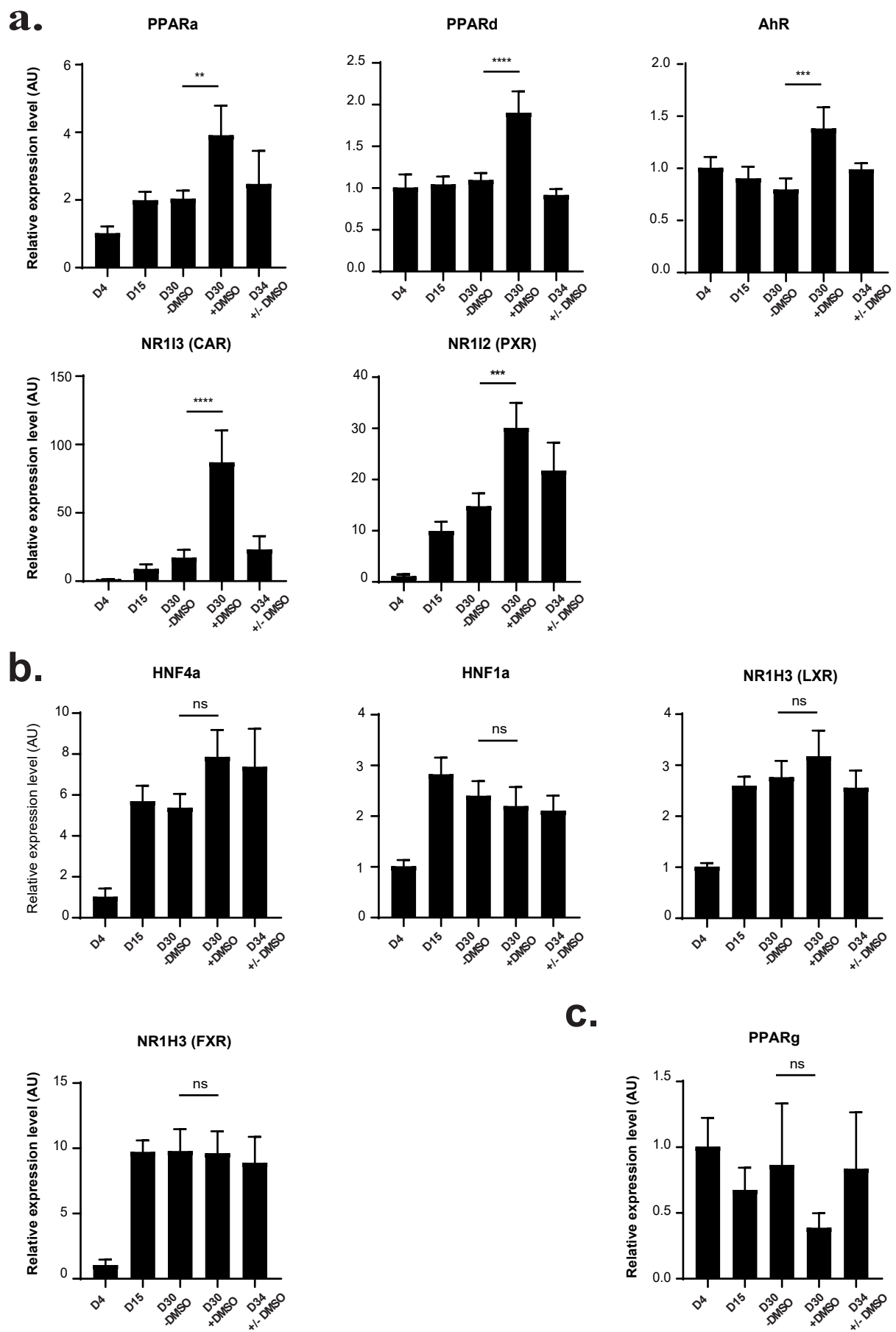

Figure S2

Supplement: Supplementary file 1 [file cells-11-02298-s001.zip › FigureS2.pdf]

A

|           |         |    | mapped reads |
|-----------|---------|----|--------------|
| untreated | H3K4me1 | R1 | 42963388     |
|           |         | R2 | 64437205     |
|           | H3K4me3 | R1 | 55730989     |
|           |         | R2 | 111950681    |
|           | H3K27ac | R1 | 33226872     |
|           |         | R2 | 80339132     |
| DMSO      | H3K4me1 | R1 | 45608421     |
|           |         | R2 | 71718954     |
|           | H3K4me3 | R1 | 41048624     |
|           |         | R2 | 76624219     |
|           | H3K27ac | R1 | 46794001     |
|           |         | R2 | 81150754     |

B

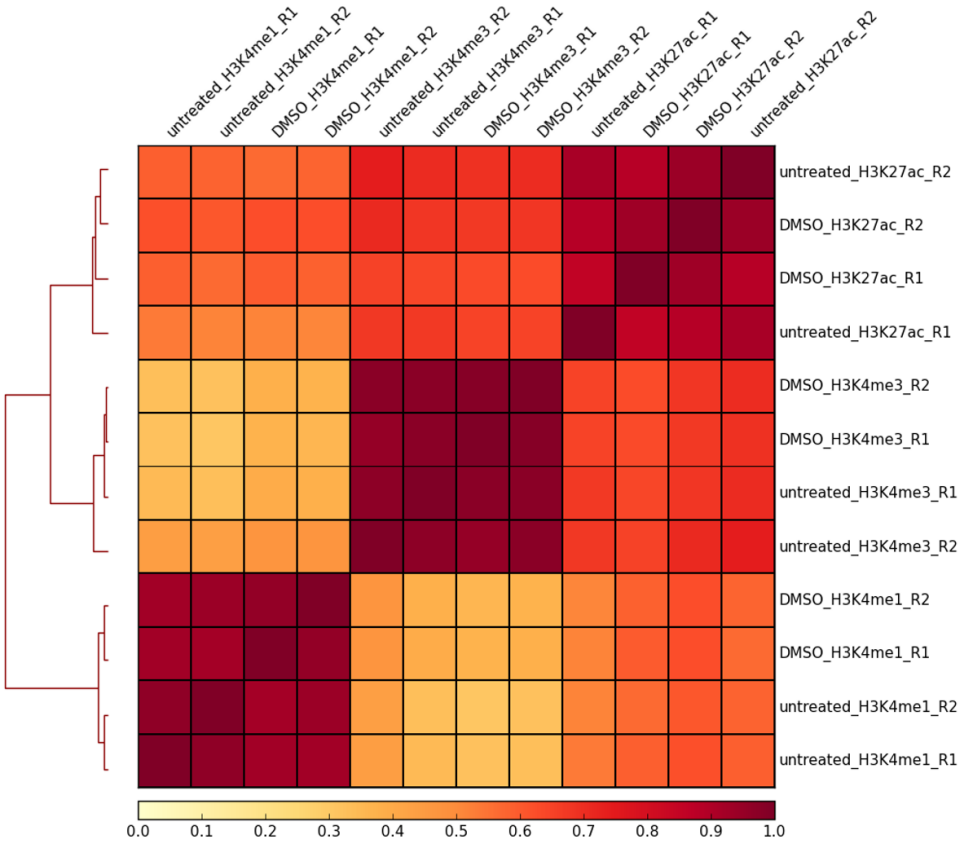

Supplement: Supplementary file 1 [file cells-11-02298-s001.zip › FigureS3.pdf]

A

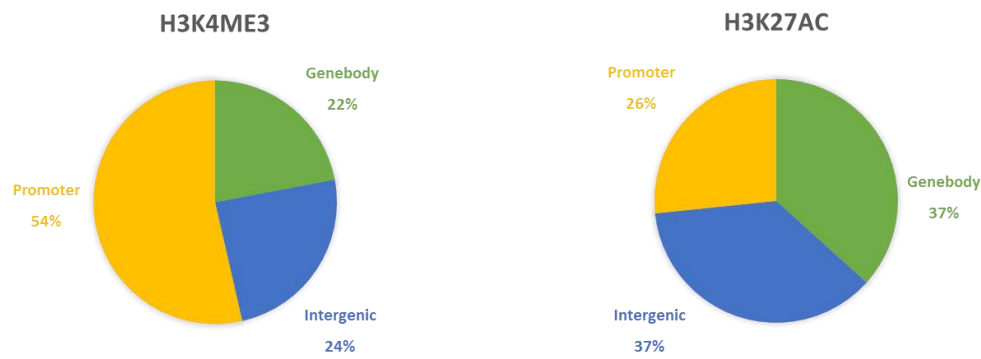

B

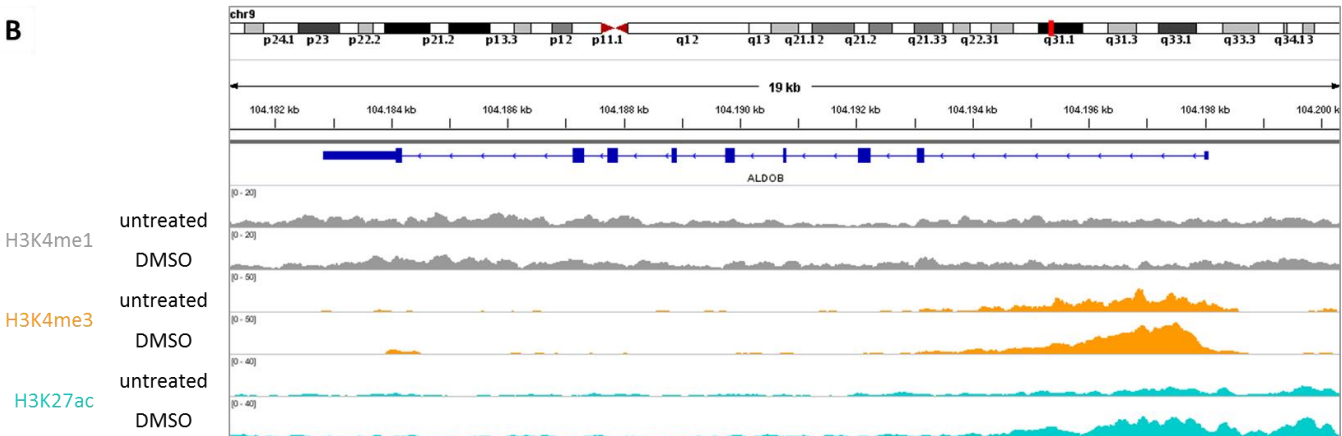

C

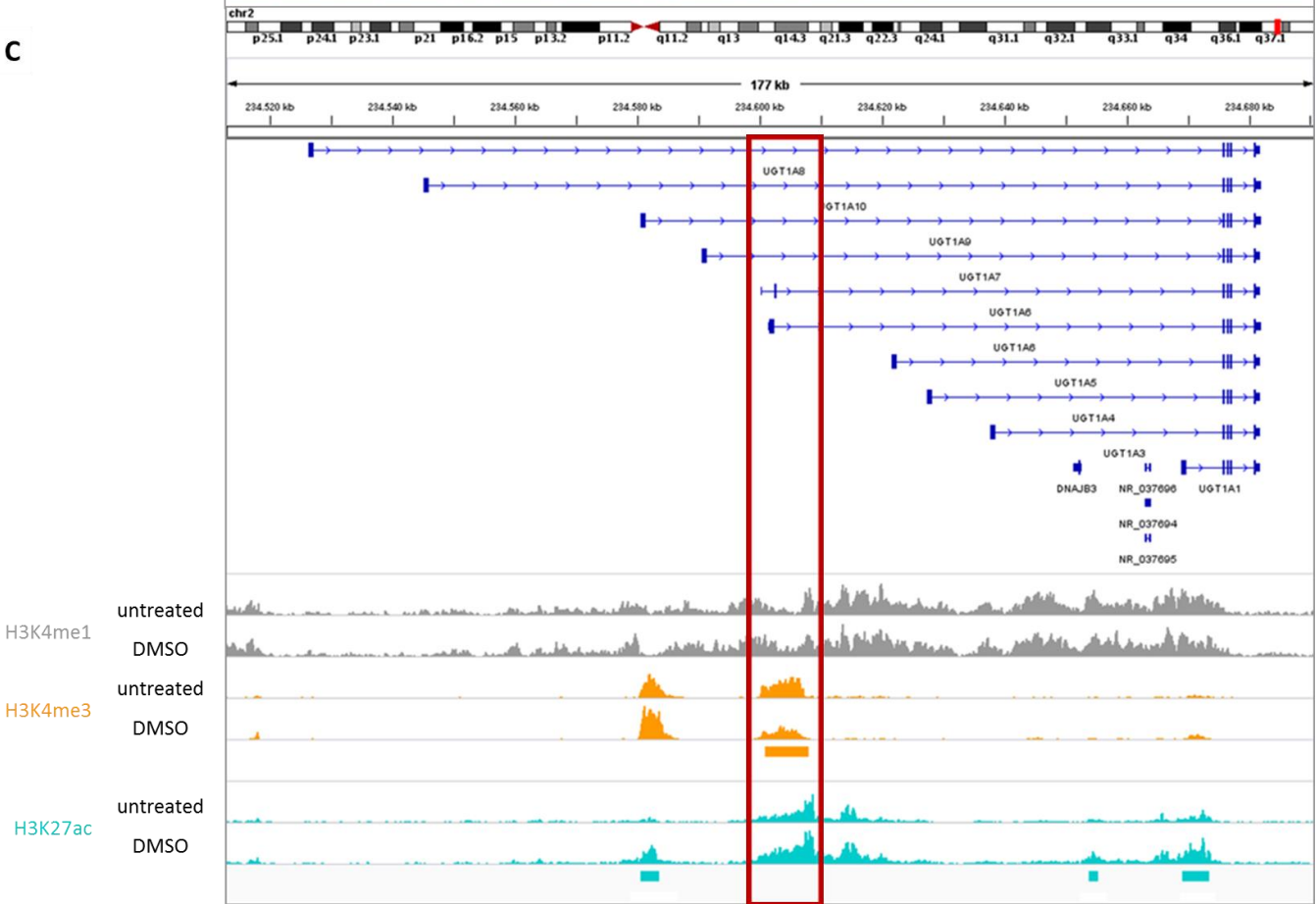

Supplement: Supplementary file 1 [file cells-11-02298-s001.zip › FigureS4.pdf]

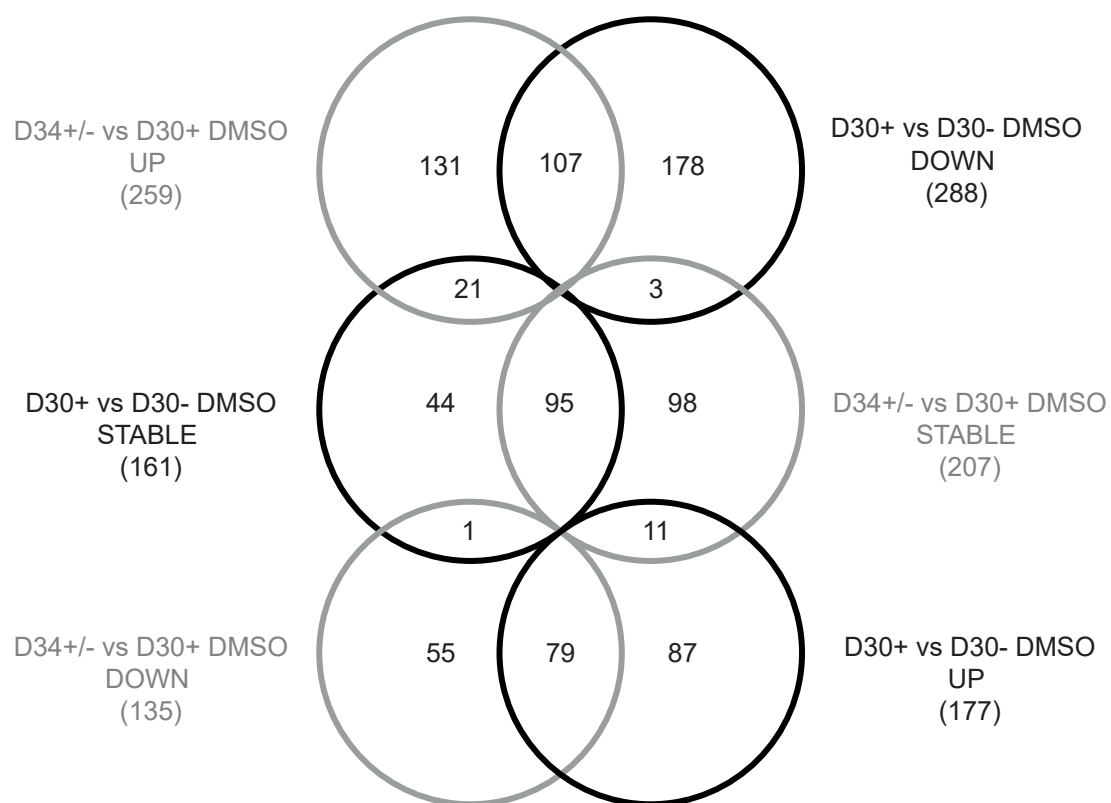

Figure S5

Supplement: Supplementary file 1 [file cells-11-02298-s001.zip › FigureS5.pdf]

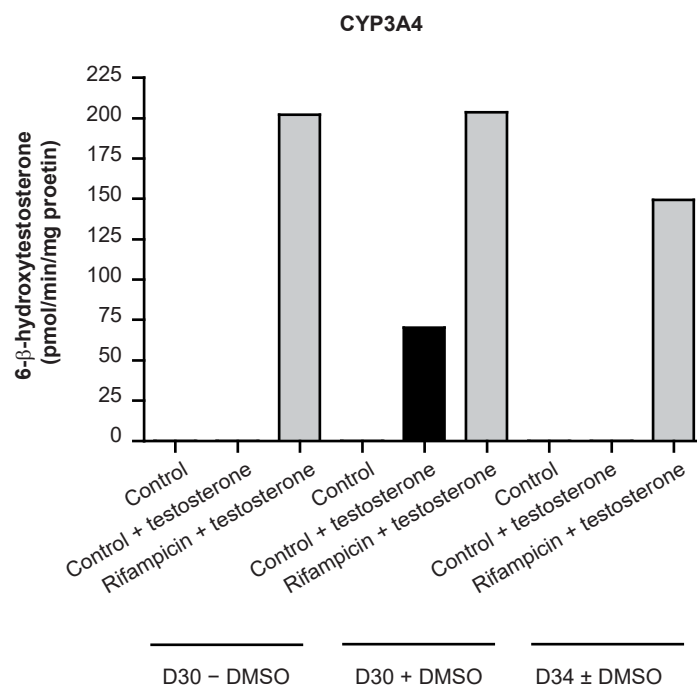

**Figure S6**

Supplement: Supplementary file 1 [file cells-11-02298-s001.zip › FigureS6.pdf]
